# pH-responsive hybrid platelet membrane-coated nanobomb with deep tumor penetration ability and enhanced cancer thermal/chemodynamic therapy

**DOI:** 10.7150/thno.68996

**Published:** 2022-05-16

**Authors:** Huang Yang, Yuan Ding, Zongrui Tong, Xiaohui Qian, Hao Xu, Fenghao Lin, Guoping Sheng, Liangjie Hong, Weilin Wang, Zhengwei Mao

**Affiliations:** 1 MOE Key Laboratory of Macromolecular Synthesis and Functionalization Department of Polymer Science and Engineering, Zhejiang University, Hangzhou 310027, China.; 2 Department of Hepatobiliary and Pancreatic Surgery, the Second Affiliated Hospital, School of Medicine, Zhejiang University, Hangzhou 310009, China.; 3 Department of Infectious Disease, Shulan (Hangzhou) Hospital Affiliated to Zhejiang Shuren University, Shulan International Medical College, Hangzhou 310022, China.

**Keywords:** platelet membrane, hybrid cell membrane, nanodots, tumor penetration, combined therapy

## Abstract

**Background:** Despite their outstanding properties in high surface-to-volume ratio and deep penetration, the application of ultrasmall nanoparticles for tumor theranostics remains limited because of their dissatisfied targeting performance and short blood circulation lifetime. Various synthetic materials with complex structures have been prepared as a multifunctional platform for loading ultrasmall nanoparticles. However, their use in nanomedicine is restricted because of unknown metabolic processes and potential physiological toxicity. Therefore, versatile and biocompatible nanoplatforms need to be designed through a simple yet effective method for realizing specific delivery and responsible release of ultrasmall nanoparticles.

**Methods:** Iron-gallic acid coordination polymer nanodots (FeCNDs) exhibits outstanding photothermal ability and Fenton catalytic performance, which can be applied for tumor inhibition via hyperthermia and reactive oxygen species. A pH-responsive platelet-based hybrid membrane (pH-HCM) was prepared via co-extrusion and acted as a safe nanoplatform to load FeCNDs (pH-HCM@FeCNDs). Subsequently, their responsive performance and penetration ability were valued considering the multicellular sphere (MCS) model in an acidic or neutral environment. Thereafter, *in vivo* fluorescence image was performed to assess targeting capability of pH-HCM@FeCNDs. Finally, the corresponding antitumor and antimetastatic effects on orthotropic breast cancer were investigated.

**Results:** In 4T1 MCS model, pH-HCM@FeCNDs group exhibited higher penetration efficiency (72.84%) than its non-responsive counterparts (17.77%) under an acidic environment. Moreover, the fluorescence intensity in pH-HCM@FeCNDs group was 3.18 times higher than that in group without targeting performance in the *in vivo* fluorescence image experiment. Finally, through *in vivo* experiments, pH-HCM@FeCNDs was confirmed to exhibit the best antitumor effect (90.33% tumor reduction) and antimetastatic effects (only 0.29% tumor coverage) on orthotropic breast cancer.

**Conclusions:** Hybrid cell membrane was an ideal nanoplatform to deliver nanodots because of its good responsibility, satisfactory targeting ability, and excellent biocompatibility. Consequently, this study provides novel insights into the delivery and release of nanodots in a simple but effect method.

## Introduction

Ultrasmall nanoparticles (1-10 nm) have garnered the attention of researchers in cancer theranostics because of their higher surface-to-volume ratio and better tissue penetration ability compared with moderate-sized nanoparticles (50-200 nm) 1-6. For instance, ultrasmall gold nanoparticles have been extensively studied in magnetic resonance imaging (MRI), photoacoustic imaging (PAI), positron emission tomography (PET), and radiotherapy owing to their high penetration ability in tumor tissue 7-9.

However, various disadvantages restrict the application of ultrasmall nanoparticles in tumor theranostics, such as low enhanced permeability and retention (EPR) effect as well as short lifetime of blood circulation 10. Therefore, it is necessary to design versatile and biocompatible nanoplatforms for realizing specific delivery and responsible release of ultrasmall nanoparticles. In addition, these nanoplatforms must possess the ability to act as large-sized nanoparticles for high accumulation while behaving as small-sized nanoparticles for deep penetration 11-15. Nanoparticles ranging from 50 to 100 nm in diameter exhibit high tumor accumulation because of quick renal clearance available to the smaller nanoparticles and difficulty in transportation in interendothelial gaps of tumor vasculature in case of the larger ones. For instance, Shen group reported that the micelles of 100 nm, composed of amphiphilic block copolymers of 7-ethyl-10-hydroxylcamptothecin (SN38) prodrug, could accumulate in tumors best with sizes ranging from 35 and 150 nm 15. Moreover, in case of nanoparticles smaller than 20 nm, penetration can be deeper than the larger ones owing to the dense extracellular matrix of tumor 11.

Several studies have focused on preparing a multifunctional platform for loading ultrasmall nanoparticles 16, 17. Applying solid or porous nanoparticles to load nanodots is a typical strategy, such as hyaluronidase-responsive hyaluronic acid, proteinase-responsive gelatin, and biodegradable dendritic silica nanoparticles 18-20. However, most of these materials exhibit minor loading efficiency because of low porosity. Moreover, the complete breakdown of these nanoplatforms is a prerequisite for the complete release of cargo, resulting in weak responsiveness. Another method involves encapsulating ultrasmall nanoparticles into responsive vesicles 21, 22. Consequently, high loading capacity can be achieved because of the large cavity in shell materials, and sensitive release can be accomplished owing to the thin outer layer with responsiveness. Various vesicles have been used as nanoplatforms to encapsulate ultrasmall nanoparticles, such as amphiphilic poly(ethyleneglycol)-poly(2-(hexamethyleneimino)ethyl methacrylate) (PEG-PC7A) and poly(ethylene glycol)-b-((2, 5-bis[(4-car-boxylicpiperidylamino)thiophenyl]-croconine)-co-4-vinyl pyridine (PEG-PCRVP) 23-27. However, it is restricted to apply synthetic materials with complex structures and functions in nanomedicine because of unknown metabolic processes and potential physiological toxicity. Moreover, in case of conventional liposomes, composed of natural phospholipids, satisfying the requirements of specific delivery and responsive release is challenging owing to lack of targeting and responsive components 28.

Biomimetic nanoplatforms based on cell membranes are widely used in nanoparticles coating because of their outstanding biocompatibility, long blood circulation, targeting performance, and immuno-stimulating capabilities 29-36. For instance, it is possible to extend the lifetime of nanoparticles' blood circulation by coating them with red blood cell membranes 30. Furthermore, engineered cell membrane-based biomimetic nanomaterials have been developed to expand the application of cell membranes in recent years 37-42. For instance, a red blood cell membrane modified with a targeting peptide on the surface was endowed with the capability of active targeting.^43^ Similarly, hybrid cell membranes of cancer cells and white blood cells possess outstanding recognition abilities for circulating tumor cells (CTCs) 44. However, their application remained limited for cell membrane-camouflaged nanoclusters to release nanodots with responsive performance.

The pH value of tumor environment (6.5-7.0) is slightly lower than that of normal tissues owing to lactic acid accumulation caused by the Warburg effect. In addition, the pH value reduces from 6.5 to 4.5 when nanomedicine undergoes early endosome (6.0-6.5), late endosome (4.5-5.5) and lysosomes (4.5-5.5) 45, 46. Consequently, the designing of pH-sensitive carriers has been studied widely for improving cellular uptake, promoting lysosomal escape, achieving specific release, enhancing penetration efficiency, and increasing retention level of nanoparticles.

Fe-based nanomaterials have been used as theranostic nanoplatform of cancer because of their outstanding catalytic performance, remarkable magnetic resonance imaging (MRI) capability, and desirable biosafety 47. During the last decades, various iron-based materials have been developed by researchers, such as iron oxide nanoparticles (ION), iron sulfide nanoparticles, ferrocene nanoparticles, iron-based metal polyphenol networks (MPNs), and iron-based metal organic frameworks (MOFs) 48. Iron-gallic acid (GA) coordination polymer nanodots (FeCNDs) were chosen in this research owing to good photothermal ability, Fenton catalytic performance, responsive MRI capability, and rapid renal clearance in tumor theranostics 49-51. Further, Fe(III) and GA have been evaluated as generally recognized as safe (GRAS) by the U.S. Food and Drug Administration (FDA) 52.

**Figure 1:**
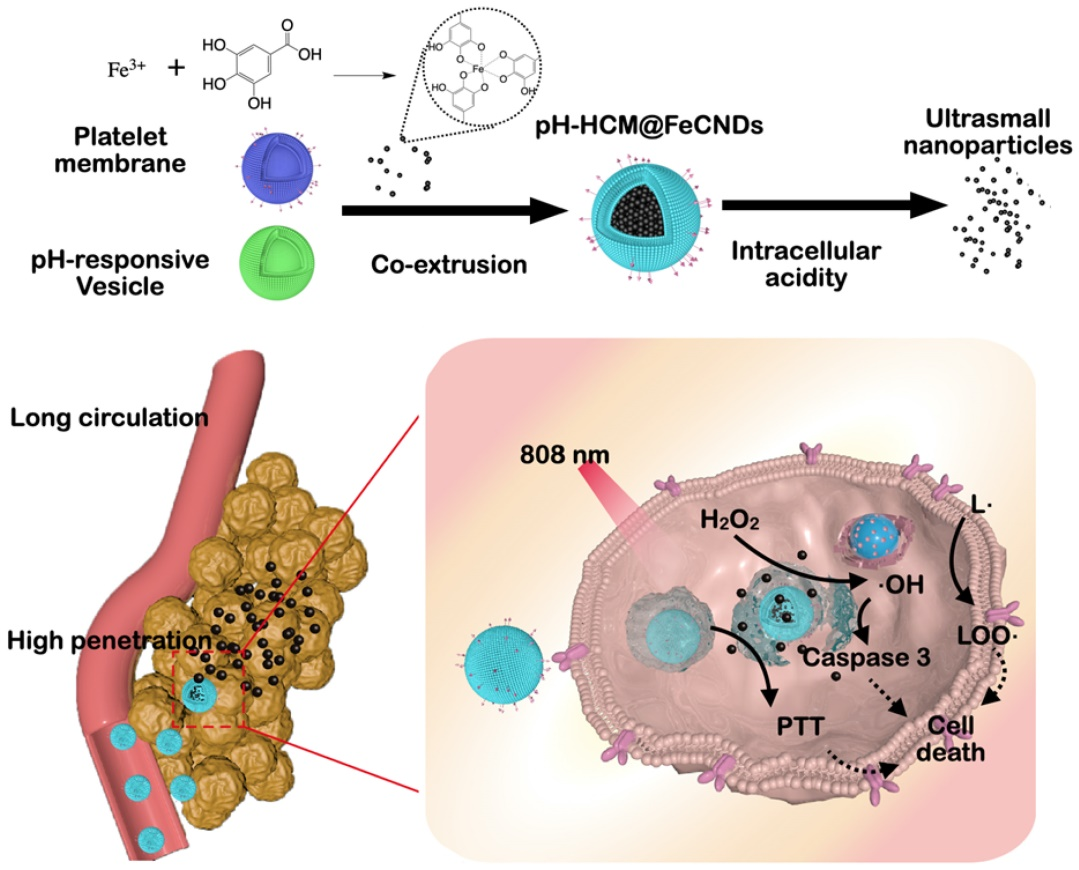
Schematic illustration for the preparation and function of pH-HCM@FeCNDs. pH-HCM@FeCNDs were prepared via co-extrusion and released FeCNDs in endosome and lysosomes. Consequently, pH-HCM@FeCNDs could penetrate into deep areas and ablate tumors effectually via thermal-chemodynamic therapy.

In this study, a pH-responsive hybrid cell membrane (pH-HCM) was prepared using platelet membranes (CM) and pH-responsive vesicles (pH-Vs) and wrapped up with FeCNDs to obtain biomimetic nanoclusters, named as pH-HCM@FeCNDs (Figure 1). CD44 receptor is a type of transmembrane glycoprotein, and involved in adhesion between cells or between cells and extracellular matrix 53. It is over-expressed on a 4T1 tumor cell membrane and significantly influences the development and metastasis of cancers. CD62p on platelet membrane can bind to CD44 receptor specificly, resulting in active targeting of pH-HCM@FeCNDs 54, 55. In addition, the pH-Vs have been merged with the CM via co-extrusion to release cargo specifically in tumor 56. The biomimetic nanocluster pH-HCM@FeCNDs was found to remain stable in the blood circulation and accumulate at the tumor site by virtue of active and passive targeting capacities. Thereafter, ultrasmall nanoparticle FeCNDs was released in the acidic environment of the lysosome and they further penetrated into the deep area for combined thermal-chemodynamic therapy 57. *In vitro* 4T1 multicellular sphere (MCS) model has been established to demonstrate the penetration ability of pH-HCM@FITC-Dextran under an acidic environment. Furthermore, *in vivo* studies have confirmed the superior antitumor and antimetastatic efficiency of pH-HCM@FeCNDs with minimal biotoxicity.

## Materials and methods

### Materials and reagents

FeCl_3_·6H_2_O were purchased from Macklin; 3,4,5-Trihydroxybenzoic acid was purchased from Shanghai D&B Biological Science and Technology Co., Ltd; ACD anticoagulant was purchased from Shanghai Zhuocai Technology Co., Ltd; Tyrode's solution and red blood cell lysis buffer were purchased from Solarbio; Phenylmethylsulfonyl fluoride (PMSF), polyvinyl pyrrolidone (PVP, MW 8000), and cholesterol were purchased from Aladdin; soybean phospholipid was purchased from Shanghai Yuanye Bio-Technology Co., Ltd; 1,2-Distearoyl-sn-glycero-3-phosphoethanolamine-poly(2-ethyl-2-oxazoline) (DSPE-PEOz, MW 2000), 1,2-Distearoyl-sn-glycero-3-phosphoethanolamine-polyethylene glycol (DSPE-PEG, MW 2000) and FITC-Dextran were purchased from Xi'an Ruixi Biological Technology Co., ltd; PKH67 and PKH26 fluorescent dye were purchased from Beijing Bio Rab Technology Co. Ltd; IR780 was purchased from Alfa Aesa; Methylene blue was purchased from sigma; H_2_O_2_ was purchased from Sinopharm Chemical Reagent Co., Ltd; BCA kit was purchased from Beyotime Biotechnology; and Cell Counting Kit-8 (CCK-8) was purchased from TargetMol, USA.

### Preparation of platelet cell membrane (CM)

The processes for separation of platelets and the preparation of hybrid cell membranes were slightly modified according to the experimental method of the Nie group 56. 15 mL of fresh blood was obtained from the eyes of thirty mice and collected in tube containing EDTA, followed by the addition of 1.5 mL of ACD anticoagulant. Subsequently, the solution (100 g, 20 min) was centriguged, followed by supernatant fluid collection to obtain platelet rich plasma. Thereafter, an equal volume of red blood cell lysis buffer was added to the plasma, which was then placed at 4 °C for 10 min to break the remaining red blood cells. Subsequently, the platelets were collected by centrifuging the treated plasma (800 g, 20 min) and resuspended using 10 mL of Tyrode's solution. Second lysis and centrifugation were required if certain red blood cells still remained. 100 μl of PMSF solution (1 mM) was added to the Tyrode's solution containing platelet to protect the membrane protein. Thereafter, the platelet was lysed via repeated freezing and thawing (five times) in liquid nitrogen and a 37 °C water bath, respectively. Whereafter, the CM was prepared by centrifuged (12000 rpm, 30 min, 4 °C) and resuspended in ultrapure water. The CM was quantified based on the protein concentration using a BCA kit and diluted to 2 mg/mL of protein for the following experiments. Moreover, the solution of CM was stored at 4 and -20 °C for short-and long-term use, respectively.

### Preparation of pH-responsive hybrid platelet membrane (pH-HCM)

19.5 mg of soybean phospholipid, 11.6 mg of cholesterol, and 15 mg of DSPE-PEOz (MW 2000) were dissolved in 9.22 mL of chloroform. Then, 1 mL of the above solution and 4 mL of chloroform were added into a round flask and evaporated slowly to form a film by rotary evaporator. Thereafter, 1 mL of ultrapure water was added to the round flask and sonicated for 20 min in an ice water bath to obtain pH-responsive liposome vesicles (pH-Vs). Different proportion of proteins on CM and pH-Vs (1: 1, 1: 2, 1: 5, and 1: 10, m/m) were obtained depending on different mass of pH-Vs added to the CM solution. Subsequently, the mixed solution was sonicated in an ice water bath for 20 min and respectively extruded through the 400, 200, and 100 nm of filter membranes via a liposome extruder. Simultaneously, DSPE-PEG (MW 2000) was used to replace DSPE-PEOz (MW 2000) to prepare the non-responsive hybrid cell membrane (npH-HCM).

### Characterization of pH-HCM

Colocalization of fluorescent-labeled pH-Vs and CM was considered for characterizing the merging of two vesicles. PKH67 fluorescent dye (green, E_x_/E_m_ = 409/502 nm) was added to the pH-Vs solution and thereafter placed at 37 °C for 5 min and 4 °C for 15 min according to the instruction. Thereafter, the solution was purified via centrifugation (12000 rpm, 12 min) to obtain PKH67-labeled pH-Vs, which was called pH-Vs (PKH67). Similarly, CM (PKH26) were obtained through the incubation of CM and PKH26 fluorescent dye (red, E_x_/E_m_ = 551/567 nm) at 37 °C for 5 min and purification via centrifugation (12000 rpm, 30 min, 4 °C). The solution of pH-Vs (PKH67) and CM (PKH26) was mixed and extruded repeatedly into a 400 nm filter membrane via a liposome extruder. Furthermore, confocal laser scanning microscope was used to confirm whether they fused after extrusion or not.

The surface electrical properties of pH-HCM prepared by different CM and pH-Vs ratios were characterized by zeta potential. The size and shape of pH-HCM were analyzed through dynamic laser light scattering (DLS) measurements and transmission electron microscopy (TEM). Further, the component of pH-HCM was characterized by infrared spectrum, and the functional proteins (CD41, CD47, integrin α6, CD62p) were detected via the conduction of the sodium dodecyl sulfate polyacrylamide gel electrophoresis (SDS-PAGE) analysis and western blot (WB).

### Preparation of pH-HCM@FeCNDs

0.2 mL of FeCl_3_·6H_2_O (100 mg/mL) aqueous solution was added to 8.8 mL of PVP (7.5 mg/mL MW 8000) aqueous solution and stirred at room temperature for 1 h. Thereafter, 1 mL of 3,4,5-trihydroxybenzoic acid (GA, 10 mg/mL) aqueous solution was added dropwise to the above solution and stirred overnight 49. Subsequently, nanodots, FeCNDs, were prepared following purification by dialysis (MWCO 14000 Da). The mass concentration of FeCNDs was obtained by freeze-drying and weighing, and the concentration of Fe in FeCNDs was measured by ICP-MS, which was approximately 3.19%. Moreover, their size and shape were characterized via DLS and TEM.

The different mass rates of pH-HCM and FeCNDs (1:0.5, 1:1, 1:1.5) were mixed and sonicated in an ice-water bath for 20 min. Thereafter, the solution was extruded through 400, 200 and 100 nm of filter membrane via liposome extruder. Subsequently, the pure nanoparticle pH-HCM@FeCNDs was obtained by dialysis (MWCO 300 KD). The discrepancy of pH-HCM@FeCNDs at pH 5.0 and 7.4 was characterized by DLS, TEM, and Zeta potential. The loading efficiency (LE) and encapsulation efficiency (EE) of FeCNDs were calculated using the following equations:

Loading efficiency (%) = (M_Fe_/(M_all_ × 3.19%)) × 100

Encapsulation efficiency (%) = (M_(Fe)_/( M_added_ × 3.19%)) × 100

where M_Fe_ was the mass of iron in pH-HCM@FeCNDs, and detected by ICP-MS, M_all_ was the mass of pH-HCM@FeCNDs detected by freeze-drying and weighing, and M_added_ was the mass of input FeCNDs.

In a similar manner, non-responsive nanoparticles npH-HCM@FeCNDs were prepared by npH-HCM and FeCNDs (m/m, 1:1.5). Hybrid membrane loading FITC-Dextran, referred to as pH-HCM@FITC-Dextran or npH-HCM@FITC-Dextran, was prepared from (n)pH-HCM and FITC-dextran (m/m, 1:1.5), and kept at 4 °C for cell experiments. IR780 and FeCNDs loaded in pH-Vs, pH-HCM and npH-HCM were prepared with the same mass ratio (nanoplatforms: FeCNDs: IR780 = 1:1.5:0.1) and maintained at 4 °C for animal experiments.

### Photothermal and catalytic performance of pH-HCM@FeCNDs

1 mL of FeCNDs or pH-HCM@FeCNDs with different concentrations (0, 12.5, 25, 50, and 100 μg Fe/mL) was added to a 1.5 mL centrifugal tube. An infrared thermal imaging camera was used to record the temperature at different irradiation intensities (0.5, 0.8, 1.2, and 1.5 W/cm^2^) of an 808 nm laser for 10 min. Similarly, 1 mL pH-HCM@FeCNDs (25 μg Fe/mL) with/without H_2_O_2_ (10 μM) was added in to a 1.5 mL centrifugal tube, whose temperature was recorded at different irradiation intensities (1.2 W/cm^2^) of an 808 nm laser for 10 min.

Methylene blue (MB) was used to characterize the catalytic performance by detecting the production of hydroxyl radicals (•OH). A mixed aqueous solution containing FeCNDs or pH-HCM@FeCNDs (25 μg Fe/mL), H2O2 (10 mM), and MB (25 μg/mL) was placed at 37 °C for 30 min or 6 h. Subsequently, the generated hydroxyl radicals were measured via the decrease in absorption at 660 nm. Furthermore, p-Phthalic acid (TA) was also used to detect •OH. Similarly, mixed aqueous solution containing FeCNDs or pH-HCM@FeCNDs (25 μg Fe/mL), H_2_O_2_ (10 mM), and TA (1.5 mM) was placed at 37 °C for 5 min, 30 min, 1 h, or 6 h. Subsequently, the generation of hydroxyl radicals was measured via fluorescence (Ex/Em = 320/425 nm).

### pH-responsive and penetrative performance of pH-HCM@FeCNDs and pH-HCM@FITC-Dextran

The pH-responsive release of pH-HCM@FeCNDs was investigated through the detection of iron. Specifically, 2 mL pH-HCM@FeCNDs or npH-HCM@FeCNDs (300 μg Fe/mL) were packed in a dialysis bag (MWCO 300 KDa) and placed in 10 mL MOPS buffer (pH 5.0 or 7.4) at 37 °C water bath with orbital shaking at 200 rpm. At predetermined time intervals, 100 μl of MOPS buffer was removed followed by the addition of another 100 μl of MOPS buffer with the same pH. The quantity of released FeCNDs was determined by iron element detection kit.

The pH-sensitive release of pH-HCM@FITC-Dextran was investigated by monitoring the FITC-Dextran release in various solutions. Typically, 5 mL PBS containing nanomaterials pH-HCM@FITC-Dextran or npH-HCM@FITC-Dextran (FITC-Dextran concentration, 1 mg/mL) were packed in a dialysis bag (MWCO 300 KDa) and placed in 20 mL PBS (pH 5.0 or 7.4) at 37 °C water bath with orbital shaking at 200 rpm. Subsequently, at predetermined time intervals, 1 mL of PBS was removed followed by the addition of another 1 mL of fresh PBS with the same pH. The quantity of released FITC-Dextran was determined by the absorption at 495 nm in the UV-vis spectrum.

The penetration of pH-HCM@FITC-Dextran was studied employing the 4T1 multicellular spheres model (MCS). 96 round-bottomed antiviscosity well plate was added with 200 μl of 5% poloxamer solution per well and placed in 37 °C water bath overnight. Thereafter, the well plate was exposed to ultraviolet radiation for 30 min after the poloxamer solution was drawn out. 5 × 10^3^ of 4T1 cells and 5 × 10^3^ of 3T3 cells were added to the treated 96-well plate and cocultured for a period to obtain 4T1 multicellular spheres with a size of approximately 400 μm. 100 μl of DMEM containing pH-HCM@FITC-Dextran or npH-HCM@FITC-Dextran (FITC-Dextran concentration: 1 mg/mL) at pH 5.0 or 7.4 was added to the 96-well plate. After incubation with nanoparticles for 12 h, 4T1 multicellular sphere was washed using PBS three times and subsequently fixed with 4% paraformaldehyde for 30 min. Thereafter, 4T1 multicellular sphere was transferred from 96-well plate to glass-bottom dish lightly and observed through a confocal laser scanning microscope.

### Cellular uptake

4T1 cells were cultured in high-glycemic DMEM containing 10% FBS and 1% pancreatin in an atmosphere of 37 °C and 5% CO_2_. Thereafter, 10^4^ of 4T1 cells were added into each well of a 96-well plate and cultured for 12 h. Further, 100 μl of DMEM containing FITC-Dextran, pH-Vs@FITC-Dextran, CM@FITC-Dextran, or pH-HCM@FITC-Dextran (FITC-Dextran content: 1 mg/mL) was added into the wells. The cells were washed three times with PBS and further stained with DAPI for 15 min following 12 h of incubation. Thereafter, blue fluorescence and green fluorescence of DAPI and FITC-Dextran were observed by fluorescence microscope.

4T1 cells were cultured in high-glycemic DMEM containing 10% FBS and 1% pancreatin in an atmosphere of 37 °C and 5% CO_2_. Subsequently, 10^5^ of 4T1 cells were added into each well of a 12-well plate. This was followed by the incubation of 1 mL of DMEM containing FeCNDs and pH-HCM@FeCNDs with same Fe concentration (25 μg/mL) with 4T1 cells for 12 h. After co-incubation, 4T1 cells were washed with PBS three times and iron content was measured by iron element detection kit.

### *In vitro* chemodynamic and antitumor efficacy

DCFH-DA fluorescent probe, which is non-fluorescent in the reduced state but a fluorescent compound DCF when the reactive oxygen species (ROS) is generated, was used to detect ROS in tumor cells. Moreover, 10^4^ 4T1 cells were incubated with 100 μl of FeCNDs and pH-HCM@FeCNDs (25 μg Fe/mL) for 8 h. After washing three times with PBS, the cells were co-cultured with DCFH-DA(10 μM) for 20 min. Thereafter, the medium was carefully removed, and the cells were washed with PBS for three times. Next, the nuclei of the tumor cell were stained with DAPI (10 μg/mL) for 20 min. Finally, ROS was observed via a fluorescence microscope, as indicated by green fluorescence.

A mixture solution of Calcein-AM (2 μM) and PI (4.5 μM) in PBS was used to stain cells to distinguish dead and live cells treated with FeCNDs or pH-HCM@FeCNDs (25 μg Fe/mL) under the 808 nm laser irradiation (1.2 W/cm^2^, 6 min). Dead cells were stained with PI and emitted red fluorescence, while live ones were stained with Calcein-AM to exhibit green fluorescence. Furthermore, 4T1 cells co-cultured with FeCNDs or pH-HCM@FeCNDs in dark were used as single therapy groups.

The CCK-8 kit was used to analyze the antitumor effect of different formulations with or without the 808 nm laser irradiation. The 4T1 cells were seeded in a 96-well plate (10^4^ cells per well) for 12 h. Thereafter, DMEM containing different concentrations of FeCNDs or pH-HCM@FeCNDs (0, 5, 10, 15, 20, and 25 μg/mL) were added to the well for 4 h. Next, the cells were irradiated with an 808 nm laser (1.2 W/cm^2^) for 6 min. Following 12 h of co-culture cells and materials, 4T1 cells were washed with PBS for three times and incubated in 100 μl of DMEM containing CCK-8 for 2 h. A microplate reader was applied to measure the absorbance at 450 nm of each well.

Flow cytometry assays were further performed to investigate thermal-chemodynamic cytotoxicity. 4T1 cells were seeded in a 6-well plate in a density of 2 × 10^5^ cells per well. The medium was replaced with a fresh medium containing FeCNDs and pH-HCM@FeCNDs (25 μg Fe/mL). After 4 h incubation, an 808 nm laser at a power density of 1.2 W/cm^2^ was used to irradiate the cells for 6 min. Subsequently, following 12 h of coculture cells and materials, the cells were stained with Annexin-V FITC and PI, and flow cytometry was performed.

### *In vitro* hemolysis evaluation

The red blood cells were obtained from fresh whole blood containing 10% ACD buffer by centrifugation (800 g, 10 min). Thereafter, the red blood cells were washed three times and diluted to 10% (v/v) with PBS. A 100 μl of PBS containing FeCNDs or pH-FeCNDs with different concentrations was added to 900 μl of the blood cells (10%, v/v), maintaining final Fe concentrations of 10, 25, 50, 100, 200, and 300 μg Fe/mL. The mixture was incubated at 37 °C for 3 h and centrifuged at 5000 rpm for 10 min. The absorbance at 541 nm of supernatant was measured to evaluate the hemolytic activity. The hemolysis ratio was calculated using the following equation:

Hem (%) = ((A_sample_ -A_background_- A_PBS_)/(A_water_ - A_PBS_)) × 100

where A_sample_, A_water_, and A_PBS_ denote the absorbance at 541 nm of supernatant when red blood cells were incubated in the sample, distilled water, and PBS, respectively, and A_background_ denotes the absorbance at 541 nm of supernatant of aqueous solution of FeCNDs or pH-HCM@FeCNDs with same concentration.

### *In vivo* fluorescence imaging and infrared thermal imaging

Orthotopic breast cancer was established in Balb/c mice by injecting 5 × 10^5^ 4T1 cells into the fat pad. When the volume of the tumor reached 100 mm^3^, those mice were used for the following experiments. 4T1 tumor-bearing mice were i.v. injected with pH-Vs@Fe/IR, pH-HCM@Fe/IR, and npH-HCM@Fe/IR (IR780 1 mg/kg) for fluorescence imaging. *In vivo* fluorescence imaging system was used to detect the fluorescence intensity, using 784 nm as the excitation light at 3, 8, 24, and 48 h post-injection.

The *in vivo* photothermal performance of pH-HCM@FeCNDs was evaluated using infrared thermal imaging. Moreover, 4T1 tumor-bearing mice were treated with PBS, FeCNDs, pH-Vs@FeCNDs, npH-HCM@FeCNDs, and pH-HCM@FeCNDs via i.v. injection. Thermography was acquired 24 h post-injection using infrared imaging devices under the 808 nm laser irradiation at a power density of 1.2 W/cm^2^ for 6 min.

### *In vivo* antitumor efficacy of orthotopic breast cancer and inhibitory effects of lung metastasis

Orthotopic breast cancer was established in Balb/c mice by injecting 5 × 10^5^ 4T1 cells in the fat pad. When the volume of the tumor reached 100 mm^3^, these mice were used for the following experiments. The mice were randomly divided into seven groups (n = 5): (I)PBS, (II) laser irradiation (808 nm, 1.2 W/cm^2^, 6 min), (III) pH-HCM@FeCNDs (3 mg Fe /kg), (IV) FeCNDs (3 mg Fe/kg) + laser irradiation (808 nm, 1.2 W/cm^2^, 6 min), (V) pH-Vs@FeCNDs (3 mg Fe/kg) + laser irradiation (808 nm, 1.2 W/cm^2^, 6 min), (VI) npH-HCM@FeCNDs (3 mg Fe/kg) + laser irradiation (808 nm, 1.2 W/cm^2^, 6 min), and (VII) pH-HCM@FeCNDs (3 mg Fe/kg) + laser irradiation (808 nm, 1.2 W/cm^2^, 6 min). Bodyweight and tumor volume were measured two times every week. The mice were sacrificed on the 25th day post-injection, and their lung tissues were removed carefully. Subsequently, the number of metastatic nodules was measured to evaluate the inhibitory effects of lung metastasis.

### *In vivo* biosafety analysis

Healthy Balb/c mice (n = 3) were i.v. injected with PBS, FeCNDs (Fe, 3 mg/kg), and pH-HCM@FeCNDs (Fe, 3 mg/kg). At 1st, 7th and 15th day post-injection, various crucial indicators were collected from blood serum, including blood urine nitrogen (BUN), aspartate aminotransferase (AST), lactate dehydrogenase (LD), creatinine (CR), creatine kinase (CK) and alanine aminotransferase (ALT), which were tested using the manufacturer's instructions.

### Statistical Analysis

Data were expressed as mean ± standard deviation (SD). Prism software was used for statistical analysis. Asterisk (*) indicated a significant difference (* P < 0.05, ** P < 0.01, *** P < 0.001).

## Results and Discussion

### Preparation and characterization of pH-HCM@FeCNDs

**Figure 2:**
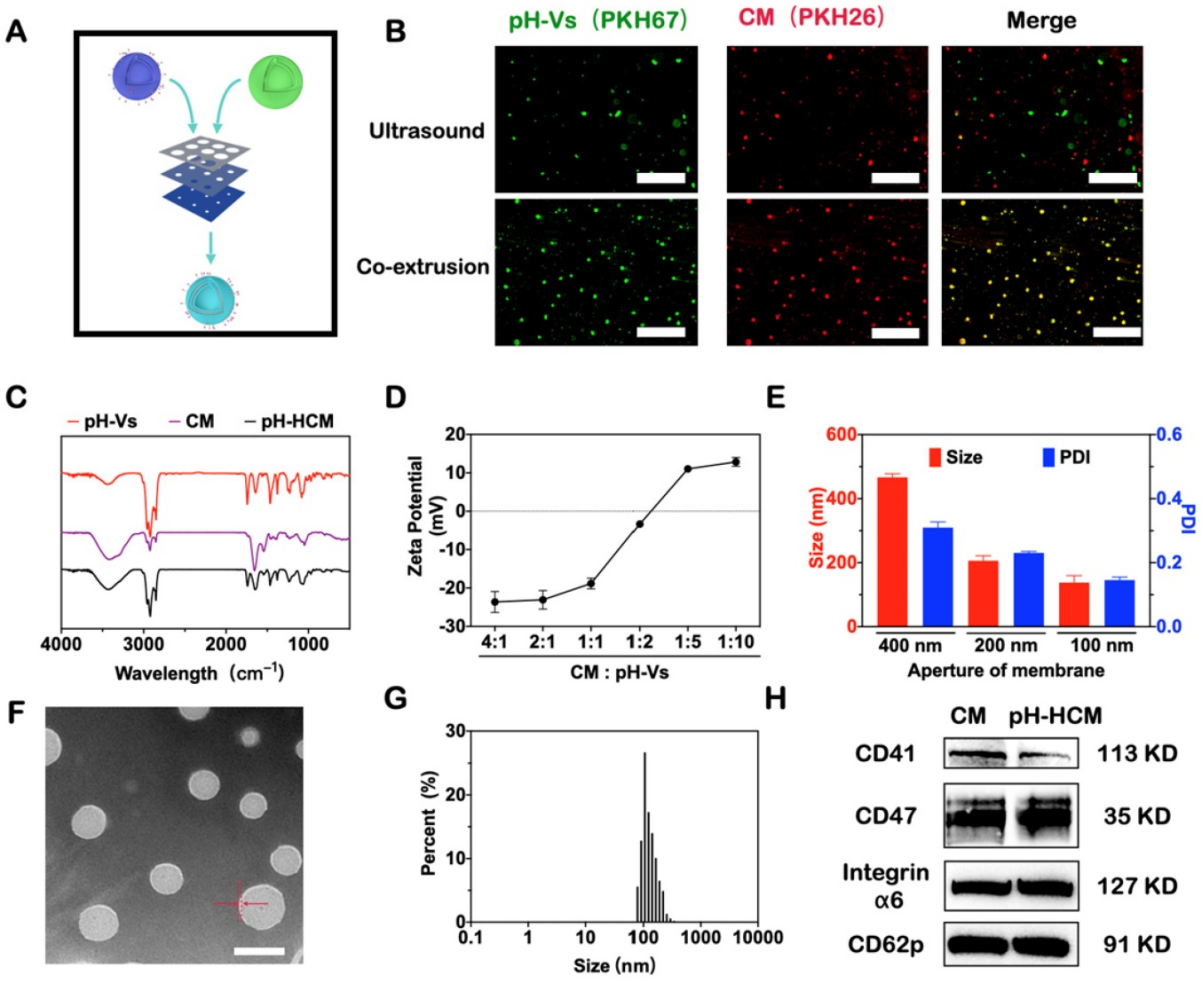
** Preparation and characterization of pH-HCM. (A)** Schematic preparation of pH-HCM. **(B)** Fluorescent pictures of production after ultrasound or co-extrusion of the mixed solution of CM (labeled with red PKH26) and pH-Vs (labeled with green PKH67). Scale bar: 10 µm. **(C)** Infrared spectra of pH-Vs, CM, and pH-HCM. **(D)** Zeta potential of pH-HCM prepared by the different mass ratio of protein on CM and pH-Vs. **(E)** Hydrodynamic size and PDI of pH-HCM during co-extrusion. **(F)** Typical TEM image of pH-HCM after staining with phosphotungstic acid. Scale bar: 100 nm. **(G)** Size distribution of the pH-HCM. **(H)** Western blot analysis of platelet-related proteins from CM and pH-HCM.

Platelet cells were collected from fresh blood via gradient centrifugation and lysis of red blood cells. Thereafter, the vesicles composed of CM were obtained via repeated freeze-thaw cycle and ultracentrifugation (12000 rpm, 30 min, and 4 °C) (Figure S1). The BCA kit was used to determine the concentration of proteins in the cell membrane. The acid-responsive lipid vesicles (pH-Vs), prepared using the filming-rehydration method, were composed of pH-responsive phospholipids (DSPE-PEOz), cholesterol, and phospholipids at a mass ratio of 0.77:0.59:1. Subsequently, the pH-Vs were fused to the CM via co-extrusion using a liposome extruder to endow the cell membrane with pH-responsiveness (Figure 2A).

To confirm the fusion of CM and pH-Vs, red fluorescent (PKH26)-labeled CM and green fluorescent (PKH67)-doped pH-Vs were used for preparing pH-HCM vesicles. As shown in Figure 2B, the co-localization of two different dyes was observed in the fluorescent image following the co-extrusion of CM and pH-Vs using 400 nm filters. However, no fusion of CM and pH-Vs was observed via ultrasound alone (Figure 2B), indicating that co-extrusion was required for the preparation of hybrid membrane. Furthermore, the bands of pH-Vs and CM at approximately 1740 and 1545 cm^-1^, respectively, were observed in the infrared spectrum of pH-HCM, indicating the fusion of pH-Vs and CM (Figure 2C).

The zeta potential of the pH-HCM, which was determined using different ratios of proteins on CM and pH-Vs, gradually changed from negative (-23.6 ± 1.1 mV) to positive (+12.8 ± 0.5 mV) with increase in the ratio of the positively charged pH-Vs (Figure 2D). The mass ratio between proteins on CM and pH-Vs was chosen at 1:1 as the preparation condition for the following experiments, considering that negatively charged nanoparticles can prevent uptake from macrophages and consequently exhibit a longer lifetime of blood circulation than the positively charged nanoparticles. The size of pH-HCM decreased stepwise from 466.6 ± 11.1 and 206.0 ± 15.4 nm to 137.4 ± 6.6 nm measured by dynamic light scattering (DLS) when they were extruded sequentially in 400, 200, and 100 nm filters (Figure 2E). In addition, the polydispersity index (PDI) also decreased from 0.31 to 0.16 during co-extrusion, indicating that nanoparticles were more homogeneous (Figure 2E). Therefore, pH-HCM with suitable size for tumor accumulation was obtained for further studies 58.

As shown in Figure 2F, the prepared pH-HCM exhibited a typical bubble structure with a thin membrane with a thickness of 7.9 nm under transmission electron microscopy (TEM), which was similar to that of CM (Figure S1). However, in a dry state, the diameter of pH-HCM ranged from 80 to 100 nm, which was smaller than that in an aqueous solution (Figure 2G, 122.4 nm). This phenomenon may be attributed to swelling effect of the vesicles and/or hydration of the surface layer.

Because proteins on CM are essential structural basis for their function, the presence of key proteins in the obtained pH-HCM must be verified. Approximately 83.8% of the proteins from CM passed to pH-HCM after extrusion, indicating the high efficiency of preparation. Subsequently, sodium dodecyl sulfate polyacrylamide gel electrophoresis (SDS-PAGE) analysis was performed to compare the protein types in CM and pH-HCM. The results (Figure S2) indicated that similar protein bands were observed between CM and pH-HCM, confirming that the majority of membrane proteins could be transferred to pH-HCM. Western blot (WB) assay was further performed to confirm the presence of key functional proteins, such as CD41, CD47, integrin α6, and CD62p, on pH-HCM (Figure 2H). Overall, the results indicated the fusion of CM and pH-Vs as well as the preservation of main proteins.

Ultrasmall FeCNDs nanodots were chosen because of their multiple antitumor effects and low physiological toxicity. As shown in Figure S3, the as-prepared FeCNDs nanodots were round nanoparticles with a mean diameter of approximately 3.5 nm. Further, the hydrodynamic diameter of the FeCNDs nanodots was approximately 4.9 nm (Figure S4), which can be attributed to the swelling effect of the outer polyvinyl pyrrolidone (PVP) layer. The iron content in the ultrasmall nanoparticles was approximately 3.2%, as detected using ICP-MS measurement. The antitumor ability of the FeCNDs nanodots was deemed to two aspects. First, Fe-based nanomaterials can catalyze hydrogen peroxide (H_2_O_2_) to produce hydroxyl radicals (•OH) and result in liposome peroxidation of tumor cells for chemodynamic therapy. Second, the FeCNDs nanodots have broad-spectrum absorption (Figure S5A), indicating a potential near-infrared (NIR) photothermal conversion agent. The photothermal curves of FeCNDs at 808 nm radiation in different Fe concentrations and laser intensities were studied (Figure S6A-B). The temperature of the FeCNDs nanodots solution (100 μg Fe/mL) increased by approximately 40 °C in 10 min under an 808 nm laser irradiation (1.2 W/cm^2^), showing outstanding photothermal conversion performance.

pH-HCM vesicles were used to load FeCNDs, named pH-HCM@FeCNDs. They were obtained via co-extrusion and purified via dialysis with a cut-off molecular weight of 300 KD. The loading and encapsulation efficiencies of the cargos were controlled by tuning the mass ratios between pH-HCM and FeCNDs. The loading efficiency (LE) was observed to gradually increase with the increase in the FeCNDs nanodots input, whereas the encapsulation efficiency (EE) gradually decreased (Figure S7). A mass ratio (pH-HCM: FeCNDs) of 1: 1.5 was used for experiments on account of the high level of LE. The mass percentage of FeCNDs nanodots in pH-HCM@FeCNDs was 39.4% based on ICP-MS result (Figure S7). Meanwhile, roughly evaluated number and volume fraction of FeCNDs nanodots in each pH-HCM on average were 4.89 × 10^3^ and 22.39%, respectively, according mass fraction, sizes, and quantity-weighted density (Table S2).

**Figure 3:**
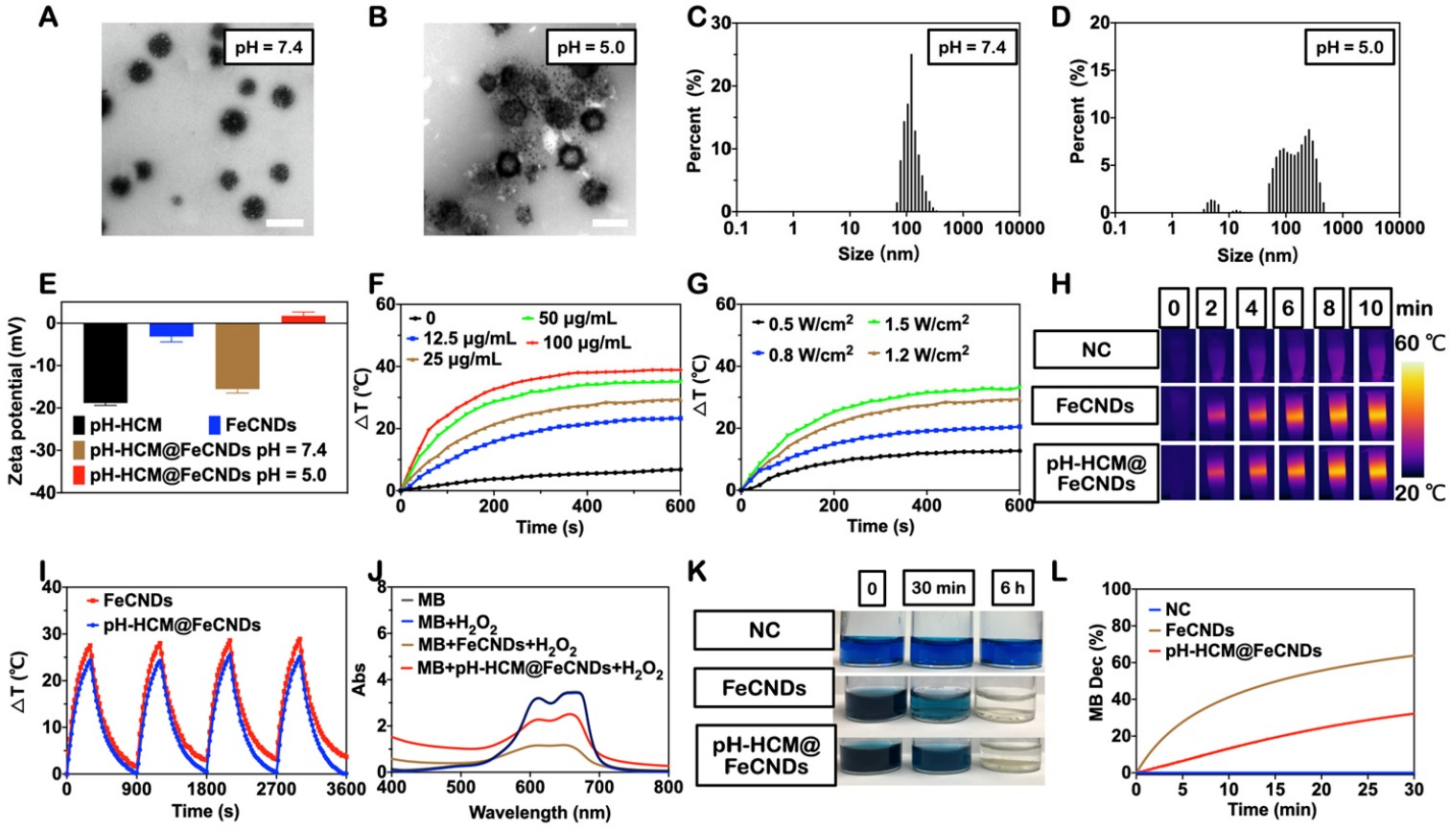
** Preparation and characterization of pH-HCM@FeCNDs.** Representative TEM images of pH-HCM@FeCNDs incubated at **(A)** pH 7.4 and **(B)** pH 5.0 for 3 h. Scale bar: 200 nm. The size distribution of pH-HCM@FeCNDs incubated at **(C)** pH 7.4 and **(D)** pH 5.0 for 3 h. **(E)** Zeta potential of different components in different pH. **(F)** Heating profiles of pH-HCM@FeCNDs with different Fe concentrations at 1.2 W/cm^2^ of the 808 nm laser irradiation. **(G)** Heating profiles of pH-HCM@FeCNDs (25 µg Fe/mL) at different power intensities of the 808 nm laser irradiation. **(H)** Infrared thermal graphics of water, FeCNDs, and pH-HCM@FeCNDs (25 µg Fe/mL) irradiated by 1.2 W/cm^2^ of the 808 nm laser for 10 min. **(I)** Photothermal stability of FeCNDs and pH-HCM@FeCNDs (25 µg Fe/mL) under 808 nm laser (1.2 W/cm^2^). FeCNDs and pH-HCM@FeCNDs were irradiated for four cycles. **(J)** UV-vis spectrum of MB solution after reaction with H_2_O_2_, H_2_O_2_ + FeCNDs, or H_2_O_2_ + pH-HCM@FeCNDs for 30 min. **(K)** Optical photos of MB solution before and after reaction with H_2_O_2_, H_2_O_2_ + FeCNDs, and H_2_O_2_ + pH-HCM@FeCNDs for 30 min and 6 h. **(L)** Time-dependent decomposition of MB by FeCNDs and pH-HCM@FeCNDs for 30 min.

As shown in Figure 3A, the obtained pH-HCM@FeCNDs vesicles were intact under neutral environment (pH = 7.4), with a diameter of approximately 130 nm, as measured from the TEM image. Their hydrodynamic diameter was 134.4 ± 44.2 nm (Figure 3C), confirming the excellent loading of the FeCNDs nanodots inside the pH-HCM vesicles. As maintaining stability in the physiological environment during circulation was crucial in case of nanomaterials, the hydrodynamic diameters and PDI of pH-HCM@FeCNDs were traced in PBS or saline overtime. The size and PDI of pH-HCM@FeCNDs exhibited no change within three days (Figure S8), indicating good stability of pH-HCM@FeCNDs under physiological conditions.

In contrast, pH-HCM@FeCNDs ruptured and released ultrasmall FeCNDs nanodots following 3 h of treatment under pH 5.0 (Figure 3B), evidenced by many small dots surrounded large vesicles. Statistically, three peaks were obtained from DLS measurement (Figure 3D, 4.8 ± 1.2, 13.5 ± 2.2, and 225.0 ± 233.7 nm) when pH-HCM@FeCNDs were treated under pH 5.0, where the peak at 4.8 nm was attributed to FeCNDs nanodots and those at 13.5 and 225.0 nm may be attributed to the reassembled nanostructures in an acidic environment. Moreover, the zeta potential changed from -15.6 mV for pH-HCM@FeCNDs under pH 7.4 to +1.7 mV for the mixture of FeCNDs and broken membrane under pH 5.0 (Figure 3E). This may be because of the release and protonation of FeCNDs (Figure S12). These results confirmed the breakage of pH-HCM@FeCNDs and release of loaded FeCNDs nanodots under pH 5.0.

To characterize the potential performance of pH-HCM@FeCNDs in tumor treatment, their photothermal and Fe-based catalytic abilities were further evaluated (Figure 2F-L). The temperature curves of pH-HCM@FeCNDs were measured under different irradiation intensities (0.5, 0.8, 1.2, and 1.5 W/cm^2^) and concentrations (0, 12.5, 25, 50, and 100 μg Fe/mL). As shown in Figure 3F-G, the temperature of pH-HCM@FeCNDs solution with 25 μg Fe/mL concentration increased by 26.9 °C under irradiation with 1.2 W/cm^2^ light intensity for 6 min, exhibiting photothermal properties similar to the FeCNDs nanodots (Figure S6). In contrast, temperature of pure water only increased by approximately 4 °C under the same conditions. Similar results were obtained using infrared thermal imaging (Figure 3H). Moreover, this photothermal performance lasted for at least four cycles without being weakened, indicating their outstanding photothermal stability (Figure 3I). Furthermore, the photothermal curve of pH-HCM@FeCNDs showed no significant change in H_2_O_2_ environment (Figure S9), indicating that the chemodynamic effect did not affect their photothermal effect. To prove the catalytic activity of pH-HCM@FeCNDs, methylene blue (MB) was used as an indicator because it could be gradually turned colorless by reaction with •OH. After incubation with pH-HCM@FeCNDs (25 μg Fe/mL) and H_2_O_2_ for 30 min, the absorbance of MB at 660 nm decreased significantly; subsequently, its color gradually changed from navy blue to light blue and eventually nearly colorless (Figure 3J, K). Typically, 32.2% of the MB was oxidized in the presence of pH-HCM@FeCNDs and H_2_O_2_. However, compared with FeCNDs, pH-HCM@FeCNDs exhibited relatively weak catalytic ability (Figure 3J-L). This could be attributed to the less FeCNDs nanodots available for H_2_O_2_ under the protection of the hybrid membrane. A similar conclusion was obtained by p-phthalic acid (TA), which reacted with •OH to generate fluorescent products (Figure S10). Overall, these results indicated that pH-HCM@FeCNDs exhibited satisfactory photothermal and catalytic capabilities for potential tumor treatment.

### Deep penetration ability of pH-HCM@FeCNDs in acid environment

A three-dimensional multicellular sphere model can better reflect the compactness of solid tumors than that with a two-dimensional cell layer. Therefore, it was an ideal model for evaluating the penetration of nanoparticles into the tumors and provided important information needed to understand the transportation pattern *in vivo*. The 4T1 MCS was prepared by coculturing 3T3 and 4T1 cells in a round-bottomed anti-adhesion well, and used for the following experiment when its diameter was approximately 400 μm. The distribution of nanoparticles inside the 4T1 MCS was observed via Z-stack scanning using a confocal laser scanning microscope (CLSM, Leica), wherein the top of MCS was recorded as 0 μm and the scanning step was set as 10 μm. FITC-Dextran was used as a model of nanodots FeCNDs for evaluating penetration efficiency because of their similar size (Figure S4, S13) and release ability (Figure S11, S12).

**Figure 4:**
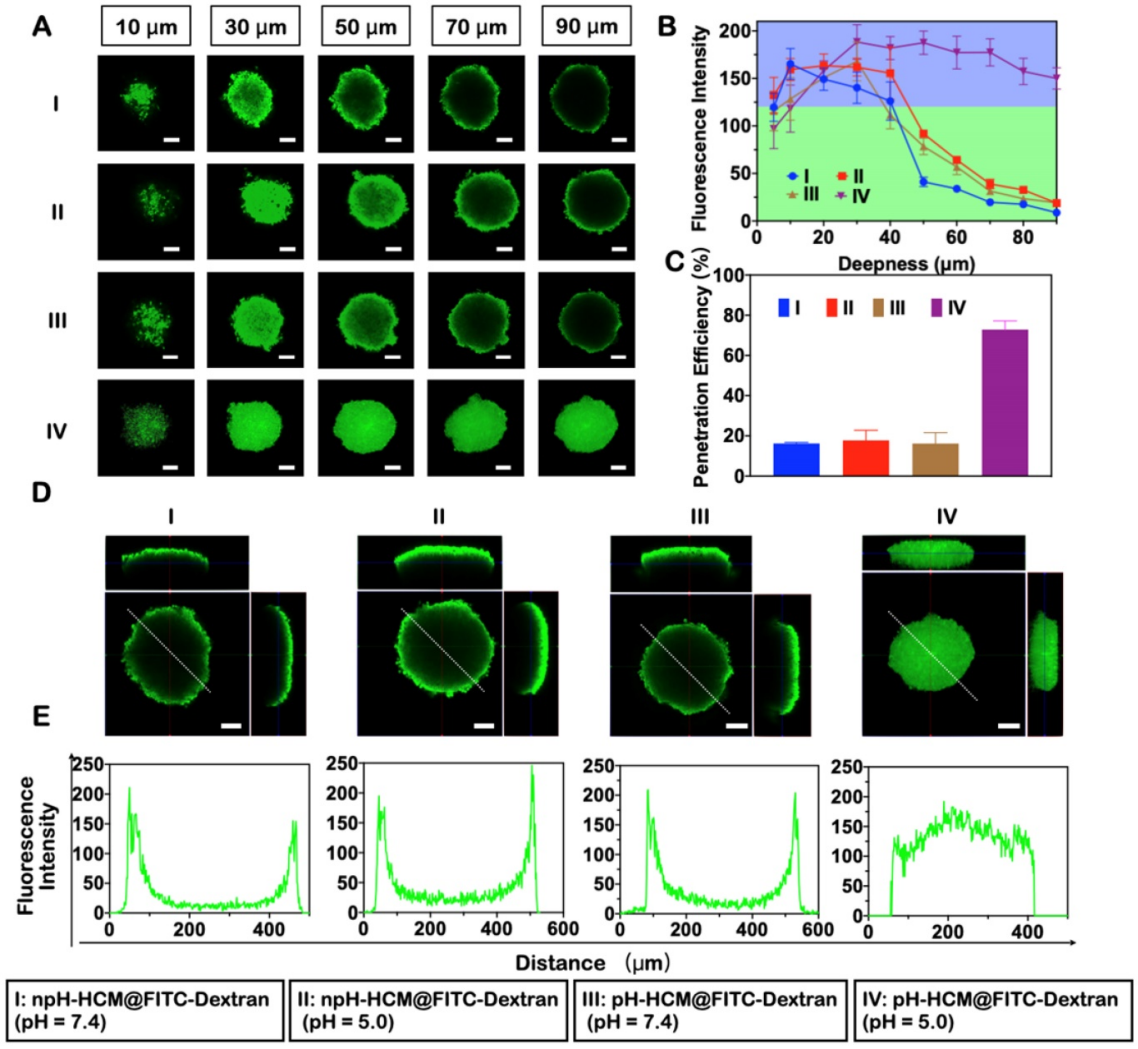
** The penetration ability of pH-HCM@FeCNDs in an acidic environment. (A)** Confocal fluorescence picture of the 4T1 multicellular spheroids in different depth after treatment with (n)pH-HCM@FITC-Dextran in pH 7.4 or 5.0 for 12 h. **(B)** Florescence intensity in cross-section center of different groups at different depths. **(C)** The penetration efficiency of the npH-HCM@FITC-Dextran and pH-HCM@FITC-Dextran in 4T1 tumor spheroids model at pH 7.4 or 5.0 for 12 h. **(D)** 3-view drawings of the cell sphere at a depth of 90 µm treated with (n)pH-HCM@FITC-Dextran in pH 7.4 or 5.0 for 12 h. **(E)** Corresponding fluorescence distribution along the white line in Figure 4D. Scale bar was 100 µm. I: npH-HCM@FITC-Dextran (pH = 7.4); II: npH-HCM@FITC-Dextran (pH = 5.0); III: pH-HCM@FITC-Dextran (pH = 7.4); IV: pH-HCM@FITC-Dextran (pH = 5.0).

4T1 MCS was treated with pH-HCM@FITC-Dextran under neutral environment (pH = 7.4) for 12 h. As Figure 4A shown, the fluorescence in the central region gradually became weaker with increasing depth. Florescence intensity in cross-section center at different depths was counted for a semi-quantitative analysis (Figure 4B). In the first 40 μm, the fluorescence intensity remained basically stable, while it decreased significantly after 40 μm (Figure 4B, group III). The penetration efficiency was counted as 16.18% (Figure 4C, group III). Meanwhile, the green fluorescence of FITC-dextran mostly appeared on surface of MCS rather than central area at the scanning depth of 90 μm (Figure 4D-E, group III). The primary reason of uneven fluorescence distribution was the large size of pH-HCM@FITC-Dextran. In contrast, green fluorescence of pH-HCM@FITC-Dextran in 4T1 MCS was observed clearly under acid environment (pH = 5.0) at the scanning depth of 90 μm (Figure 4A, group IV). The fluorescence intensity at central region remained stable on the whole (Figure 4B, group IV), and its penetration efficiency was counted as 72.84% (Figure 4C, group IV), which was more than four times that under neutral conditions. This performance of enhanced penetration was attributed to the rapid release of FITC-Dextran from pH-HCM@FITC-Dextran under acidic conditions.

Non-responsive nanoparticles npH-HCM@FITC-Dextran (Figure 4, group I and II) was prepared as a negative control of pH-HCM@FITC-Dextran. Green florescence of npH-HCM@FITC-Dextran in central area was difficult to observe in acidic or neutral conditions as the depth increased. Similar to pH-HCM@FITC-Dextran under environment of pH = 7.4, the fluorescence intensity of npH-HCM@FITC-Dextran under neutral environment (pH = 7.4) was stable before 40 μm, and depressed after 40 μm (Figure 4B, group I). The fluorescence intensity of npH-HCM@FITC-Dextran under acidic environment (pH = 5.0) was higher slightly than than under neutral environment (Figure 4B, group II), which was a result of faster release of FITC-Dextran (Figure S11A). Their penetration efficiencies were 16.24% at pH 7.4, and 17.77% at pH 5.0, respectively (Figure 4C, group I and II). According to three views and fluorescence distribution along white line, green florescence of central area was much lower than that of surface (Figure 4D-E, group I and II), which was because releasing FITC-Dextran from npH-HCM@FITC-Dextran under neutral or acidic conditions was challenging.

### *In vitro* antitumor effect of pH-HCM@FeCNDs

**Figure 5:**
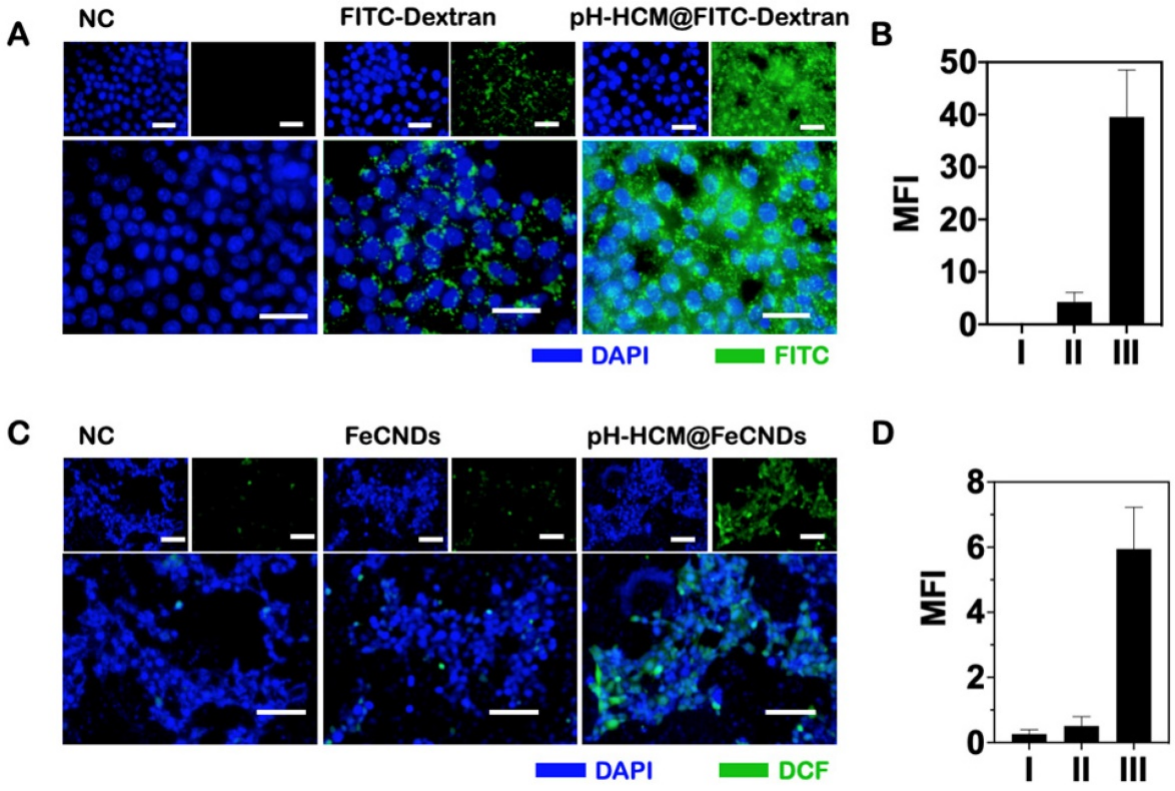
** (A)** Fluorescence images of the 4T1 cells incubated with (I) DMEM, (II) FITC-Dextran, and (III) pH-HCM@FITC-Dextran. Blue fluorescence represents nuclear from DAPI; green fluorescence represents FITC-Dextran. Scale bar: 50 µm. **(B)** Corresponding mean green fluorescence intensity of Figure 5A. **(C)** Fluorescence images of the 4T1 cells stained with DCFH-DA after incubation with (I) PBS, (II) FeCNDs, and (III) pH-HCM@FeCNDs. Blue fluorescence represents nuclear from DAPI; green fluorescence represents the level of ROS. Scale bar: 100 µm. **(D)** Corresponding mean green fluorescence intensity of Figure 5C.

pH-responsive lipid vesicles loaded with FITC-Dextran (pH-Vs@FITC-Dextran) and CM vesicles loaded with FITC-Dextran (CM@FITC-Dextran) were prepared as negative controls of pH-HCM@FITC-Dextran in cellular uptake experiment. As shown in Figure 5A, the fluorescence images indicated that FITC-Dextran loaded in the hybrid membrane were acquired more easily by the 4T1 cells than FITC-Dextran. The average fluorescence intensity of the 4T1 cells that internalized pH-HCM@FITC-Dextran was approximately 9.13 times of the cells treated with free FITC-Dextran (Figure 5B). Thus, the cause of the increased cellular uptake was further investigated using pH-Vs and CM as nanoplatforms (Figure S14A). As shown in Figure S14B, the average fluorescence intensity of the 4T1 cells treated with pH-Vs@FITC-Dextran and CM@FITC-Dextran increased by 1.72 and 3.44 times that treated using FITC-Dextran alone, respectively, indicating that CM was the primary reason for the increased cellular uptake. Moreover, when 4T1 cells were pretreated with CD62p (4T1^CD62p+^), their uptake ability for rhodamine B-labeled platelet cell membrane (CM^RB^) was lower evidently than that of 4T1 cells pretreated without CD62p (4T1^CD62p -^). This result illustrated that CD62p was an essential factor in mediating the targeting of platelet membrane-based nanomaterials to tumor cells (Figure S15).

Encouraged by enhanced pH-responsive, photothermal, and catalytic activities of pH-HCM@FeCNDs, as well as their ability of deep penetration into MCS and high uptake by tumor cells, we examined their performance against tumor cells *in vitro*. First, 2,7-dichlorodihydrofluorescein diacetate (DCFH-DA), a probe that can react with •OH to produce 2,7-dichlorofluorescein (DCF) with green fluorescence, was used to detect the intracellular level of reactive oxygen species (ROS) generated by pH-HCM@FeCNDs. As shown in Figure 5C and D, the ROS level in 4T1 cells increased by approximately 22.84 times when the cells were treated with pH-HCM@FeCNDs. In contrast, it increased by only 1.96 times when the cells were treated with FeCNDs. The significantly enhanced ROS levels of the former can be attributed to high iron uptake, which was 6.76 times of the latter (Figure S16). Meanwhile, generated ROS could result in cell death by inducing cell membrane liposome peroxidation and activation of caspase 3 (Figure S17). Second, the photothermal effect of FeCNDs and pH-HCM@FeCNDs on tumor cells was evaluated. Following treatment with FeCNDs and pH-HCM@FeCNDs (25 μg Fe/mL), and subsequently being irradiated for 6 min under the 808 nm laser (1.2 W/cm^2^), the temperature of the culture medium increased to 57.6 and 57.8 °C, respectively, exhibiting photothermal performance similar to ablate cells (Figure S18 A, B).

**Figure 6:**
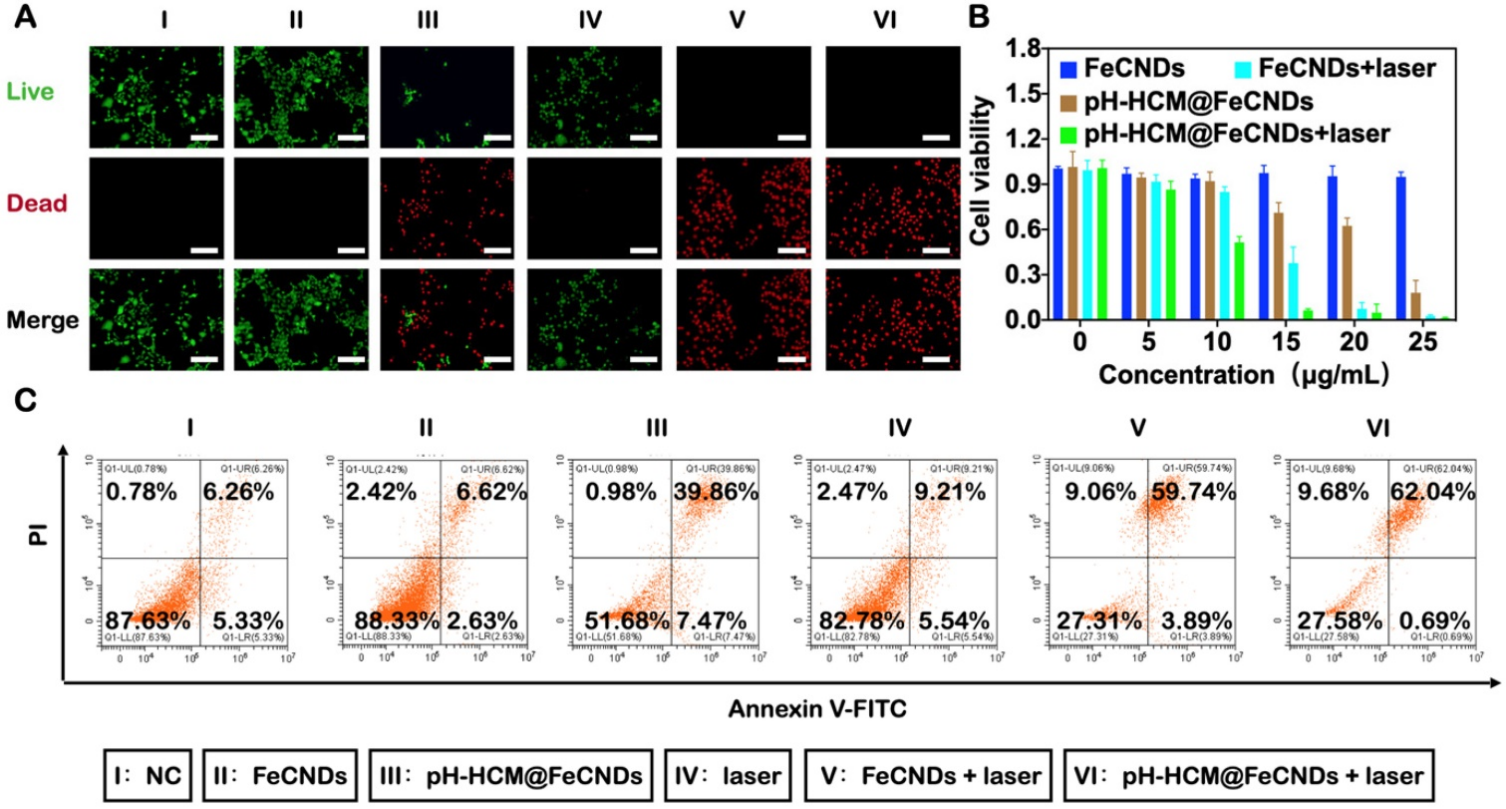
***In vitro* antitumor effect of pH-HCM@FeCNDs. (A)** Fluorescence images of the 4T1 cells stained with calcein-AM/PI after different treatment. Green fluorescence represents live cells; red fluorescence represents dead cells. Scale bar: 100 µm. **(B)** Cell viability of the 4T1 cells incubated with different concentration of FeCNDs or pH-HCM@FeCNDs for 12 h with or without the 808 nm laser irradiation (1.2 W/cm^2^, 6 min). **(C)** Flow cytometry analysis of the 4T1 cells incubated with (I) PBS, (II) FeCNDs, (III) pH-HCM@FeCNDs, (IV) PBS + laser, (V) FeCNDs + laser, and (VI) pH-HCM@FeCNDs + laser.

Calcein acetoxymethyl ester and propidium iodide (PI) were used to stain the 4T1 cells to distinguish between living and dead cells (Figure 6A) after different treatments: (I) NC, (II) FeCNDs, (III) pH-HCM@FeCNDs, (IV) laser, (V) FeCNDs + laser, and (VI) pH-HCM@FeCNDs + laser. The concentration of Fe in groups II, III, V, and VI was 25 μg/mL. pH-HCM@FeCNDs group showed satisfactory toxicity to the 4T1 cells, whereas the FeCNDs nanodots group showed negligible antitumor effect, indicating that chemodynamic therapy can be significantly enhanced significantly by target performance of pH-HCM. Furthermore, almost all the 4T1 cells were dead after treatment with FeCNDs or pH-HCM@FeCNDs under irradiation with the 808 nm laser, demonstrating effective antitumor ability by thermal/chemodynamic therapy. Further, the viability of the 4T1 cells treated with pH-HCM@FeCNDs was evaluated using the CCK-8 assay (Figure 6B). When FeCNDs nanodots were used alone, the viability of tumor cells remained as high as 94.92% although the concentration of Fe reached 25 μg/mL, showing minimal toxicity. However, the viability of 4T1 cells reduced to 18.06% when treated with 25 μg/mL pH-HCM@FeCNDs (Fe concentration), indicating an outstanding antitumor effect of pH-HCM@FeCNDs based on chemodynamic therapy. In contrast, the cell viability decreased to 2.60 and 1.26%, respectively, when the cells were treated with 25 μg/mL FeCNDs and pH-HCM@FeCNDs (Fe concentration) under irradiation with an 808-nm laser (1.2 W/cm^2^, 6 min), implying that photothermal ability was another crucial factor contributing to antitumor effect. The cell viability of 4T1 treated with pH-Vs@FeCNDs and npH-HCM@FeCNDs was further evaluated (Figure S19). *In vitro* antitumor effect of pH-HCM@FeCNDs was similar to that of npH-HCM@FeCNDs, which was better than that of pH-Vs@FeCNDs. The discrepancy in cell viability between pH-HCM@FeCNDs and pH-Vs@FeCNDs resulted from different cellular uptake (Figure S14B). Moreover, pH-HCM@FeCNDs showed relatively low toxicity to 3T3 cells, which hardly express CD44 receptors, indicating a selective toxic effect of this nanomaterial on tumor cells and normal cells (Figure S20).

To distinguish the different phases of 4T1 cells treated with various formulations, the cells were labeled with PI/Annexin V-FITC and used for flow cytometry. For the 4T1 cells treated with FeCNDs nanodots, the proportions of early apoptotic, late apoptotic, and necrotic cells were 2.63, 6.62, and 2.42%, respectively (Figure 6C and S21). However, on treatment with pH-HCM@FeCNDs, they changed to 7.47, 39.86, and 0.98%, respectively. Further, the mortality rate of the 4T1 cells increased to 71.72% (62.04 and 9.68% for late apoptotic and necrotic cells, respectively, as shown in Figure 6C and S21) for the last group, which combined photothermal and chemodynamic effects, thereby confirming the promising potential for tumor treatment.

### *In vivo* antitumor and antimetastatic effect of pH-HCM@FeCNDs

**Figure 7:**
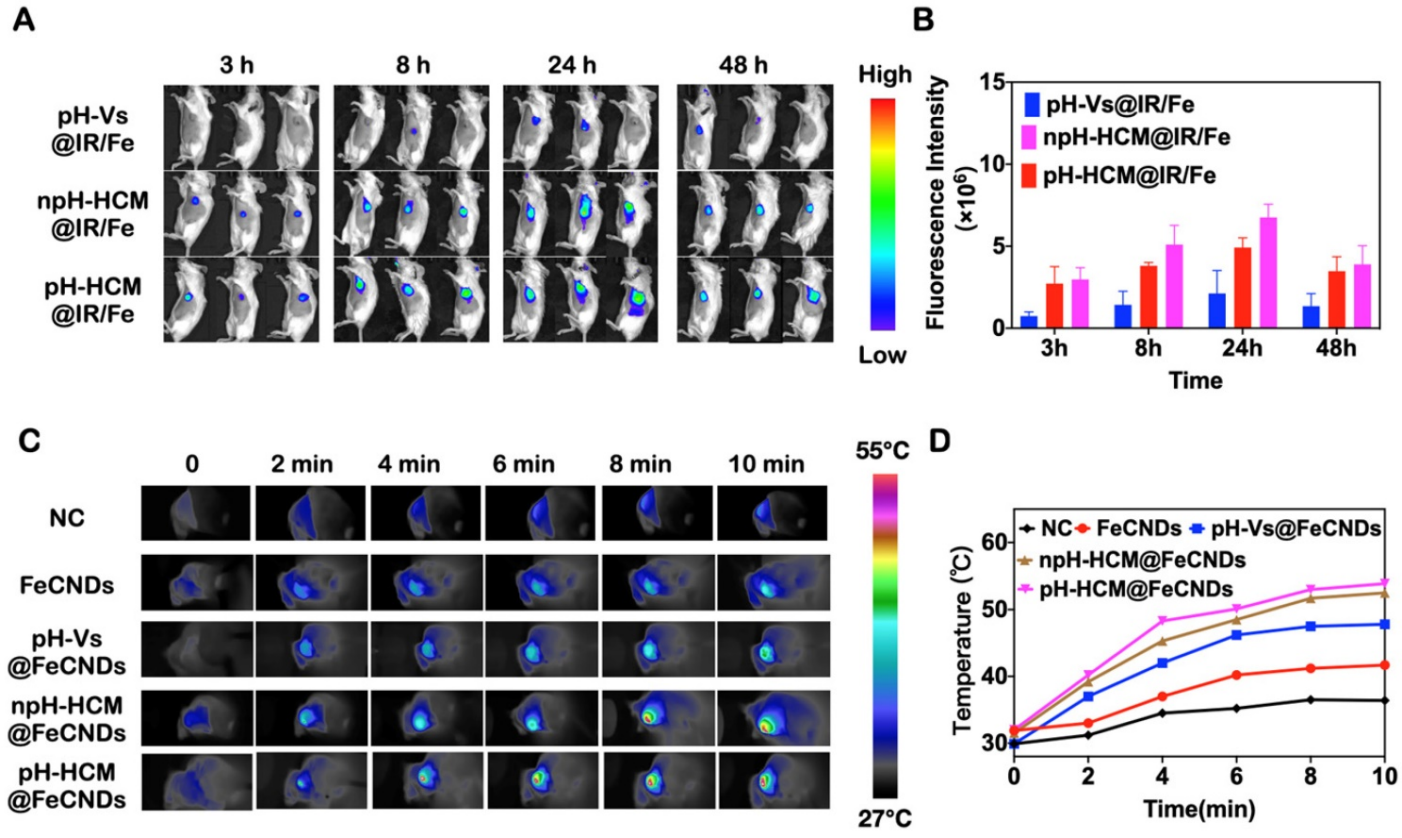
** (A)** *In vivo* fluorescence image of the 4T1 tumor-bearing mice and **(B)** fluorescence intensity of tumor at different time after i.v. injection with pH-Vs@IR/Fe, npH-HCM@IR/Fe, or pH-HCM@IR/Fe (n = 3). **(C)** Infrared thermal graphics of tumor-bearing mice under irradiation with 1.2 W/cm^2^ of 808 nm laser after injection with PBS, FeCNDs, pH-Vs@FeCNDs, npH-HCM@FeCNDs, and pH-HCM@FeCNDs. **(D)** Temperature of tumor area after different treatments.

The target ability of nanocarriers and optimal window of photothermal therapy were investigated via *in vivo* fluorescence imaging. Fluorescence molecule IR780 was loaded into pH-Vs@FeCNDs, npH-HCM@FeCNDs, and pH-HCM@FeCNDs, which were named pH-Vs@IR/Fe, npH-HCM@IR/Fe, and pH-HCM@IR/Fe, respectively. Thereafter, 4T1 tumor-bearing mice were imaged at scheduled time points post i.v. injection (1 mg IR780/kg). The average fluorescence intensity (Figure 7A, B) showed that all of pH-Vs@IR/Fe, npH-HCM@IR/Fe, and pH-HCM@IR/Fe accumulated continuously in the first 24 h; however, they began to decrease after 48 h. Therefore, the 24^th^ hour after the i.v. injection was used as a therapeutic window for photothermal treatment. Consequently, owing to the outstanding active targeting ability of the platelet membrane, the average fluorescence intensity of pH-HCM@IR/Fe was found to be more than three times that of pH-Vs@IR/Fe. Moreover, it was evident from the fluorescence photographs of tumors (Figure S22), we could also observe that fluorescence intensity of pH-HCM@FeCNDs group was significantly higher than that of pH-Vs@IR/Fe group. However, the average fluorescence intensity of pH-HCM@IR/Fe was slightly higher than that of npH-HCM@IR/Fe possibly owing to released IR780 from pH-HCM.

The *in vivo* photothermal effect before the antitumor experiment needed to be evaluated because the appropriate temperature increase was critical for photothermal therapy. As shown in Figure 7C, D, the temperature of the tumor was approximately 31.0 °C after injection with an anesthetic and 36.4 °C when irradiated with the 808 nm laser (1.2 W/cm^2^, 10 min). However, the temperature increased to 41.7, 47.8, 52.5, and 53.9 °C when 4T1 tumor-bearing mice were treated with FeCNDs + laser, pH-Vs@FeCNDs + laser, npH-HCM@FeCNDs + laser, and pH-HCM@FeCNDs + laser, respectively. Considering that the photothermal conversion efficiency of FeCNDs was not affected by the outer vesicles, the increase of tumor temperature was primaily dependent on the retention of FeCNDs in the tumor. In addition, a moderate increase of temperature in FeCNDs group was observed possibly owing to quick renal clearance. Compared with pH-Vs@FeCNDs, npH-HCM@FeCNDs and pH-HCM@FeCNDs actively targeted the tumor tissue owing to platelet membrane and caused higher increase of temperatures, showing satisfactory photothermal effect.

**Figure 8:**
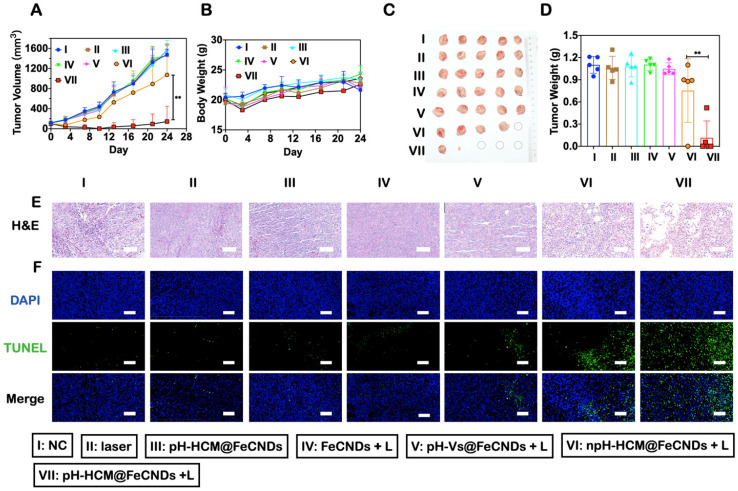
** Inhibitory effects of orthotopic breast cancer of pH-HCM@FeCNDs. (A)** Tumor growth inhibition curves and **(B)** bodyweight of the mice bearing 4T1 tumors after different treatment (n = 5, ** P < 0.01). **(C)** Photograph and **(D)** weight of orthotropic tumor harvested from the mice treated with different formulations on the 24^th^ day (n = 5, ** P < 0.01). **(E)** H&E and **(F)** TUNEL staining of tumor issues collected from the mice administrated with different formulations. Scale bar: 100 µm. The data are expressed as means ± S.D. I: PBS, II: laser, III: pH-HCM@FeCNDs, IV: FeCNDs + laser, V: pH-Vs@FeCNDs + laser, VI: npH-HCM@FeCNDs + laser, and VII: pH-HCM@FeCNDs + laser. The irradiation density was 1.2 W/cm^2^, and the irradiation time was 6 min.

After observing penetration performance, *in vitro* antitumor effect, and active targeting ability, the *in vivo* anticancer effect of pH-HCM@FeCNDs was investigated in 4T1 tumor-bearing Balb/c mice. The mice were randomly divided into seven groups when the volume of the 4T1 tumor reached 100 mm^3^: (I) PBS, (II) laser, (III) pH-HCM@FeCNDs, (IV) FeCNDs + laser, (V) pH-Vs@FeCNDs + laser, (VI) npH-HCM@FeCNDs + laser, and (VII) pH-HCM@FeCNDs + laser. As shown in Figure 8A, compared with the mice treated with PBS, little antitumor effect was observed in those treated with laser, pH-HCM@FeCNDs, FeCNDs + laser, and pH-Vs@FeCNDs + laser; while the tumor volume in the npH-HCM@FeCNDs + laser group reduced by 27.52%, demonstrating a moderate anticancer outcome. The best antitumor effect was observed in pH-HCM@FeCNDs + laser group, wherein 90.33% tumor reduction was achieved compared with the control group. Excessive differences in groups VI and VII may be a result of different tumor recurrence at early stage (Figure S23). However, the primary orthotopic tumors of three mice in this group were completely eradicated without recurrence during the treatment period owing to the responsive release of FeCNDs nanodots (Figure 8C, S12).

After treatment, the mice were sacrificed and their tumors were collected (Figure 8C). The average weight of tumors in pH-HCM@FeCNDs + laser group was 0.10 g, which was significantly lower compared to those in other groups (1.10, 1.06, 1.08, 1.11, 1.06, and 0.84 g in groups I, II, III, IV, V, and VI, respectively). This indicated that multifunctional nanoparticles with high permeability, targeting ability, and combined anticancer effect could effectively inhibit the growth of tumors (Figure 8D). Moreover, H&E and TUNEL staining of tumor sections indicated severe necrosis triggered by pH-HCM@FeCNDs (Figure 8E, F).

**Figure 9:**
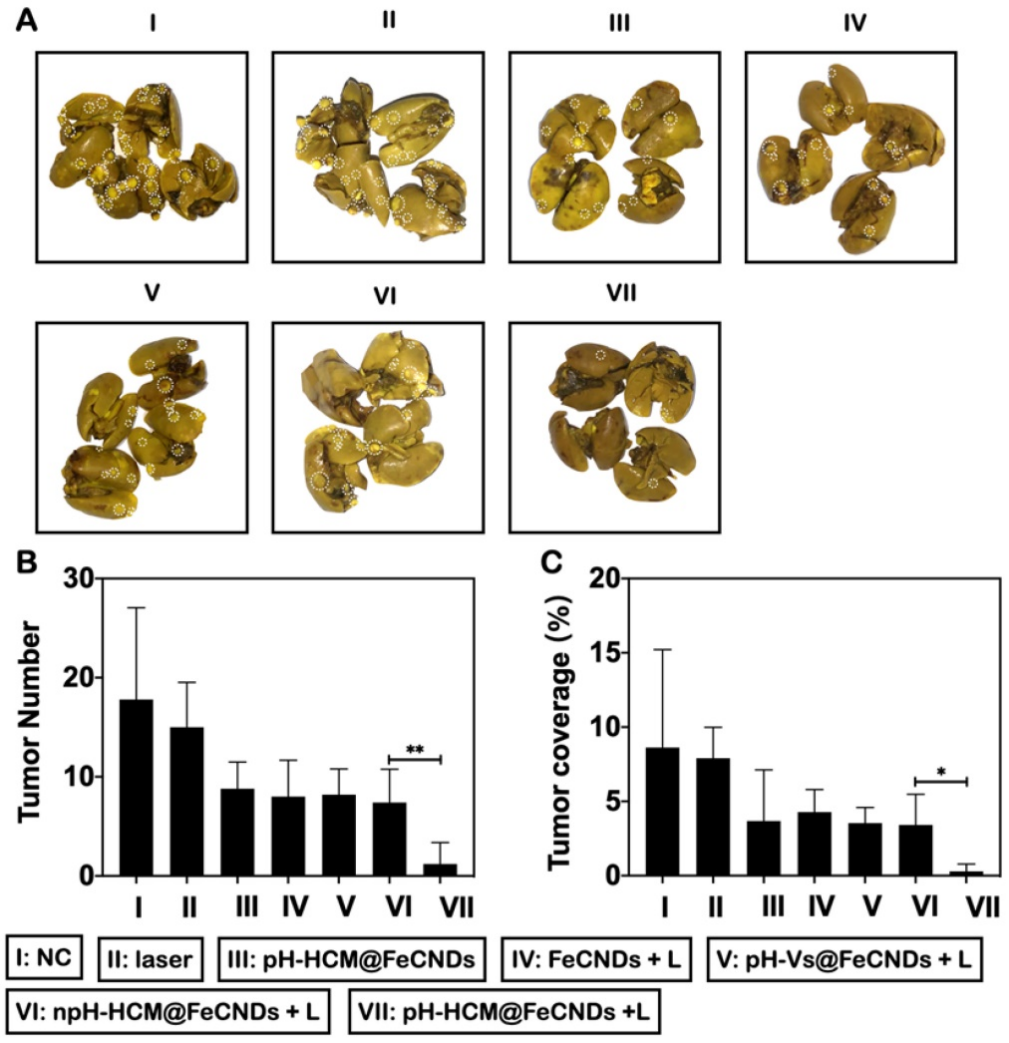
** Inhibitory effects of lung metastasis of pH-HCM@FeCNDs. (A)** Photographs of lungs in different groups. The white circles present detected metastatic nodules in lung tissue. **(B)** The number of tumor nodules and **(C)** their coverage percentage in lungs from each group (* P < 0.05, ** P < 0.01).

The antimetastatic effects of these formulations were analyzed by number and coverage rate of surface metastases in lung tissues (Figure 9A). As shown in Figure 9B, average number of metastatic nodules in lungs was 1.25 in group VII, whereas they were 17.8, 15.0, 8.8, 8.0, 8.2, and 7.4 in groups I, II, III, IV, V, and VI, respectively, implying the best antimetastatic outcome by pH-HCM@FeCNDs. Further, consistent results were obtained by analyzing the proportion of ​​ metastases to entire lung. As shown in Figure 9C, only 0.29% of lungs were occupied by tumors in group VII, which was significantly lower than other treatments (8.22, 7.42, 3.32, 3.36, 3.65, and 3.33% in groups I, II, III, IV, V, and VI, respectively). Therefore, pH-HCM@FeCNDs significantly inhibited the growth of primary tumors significantly while also limiting the lung metastasis effectively owing to the excellent responsibility and targeting ability.

### Toxicity evaluation of pH-HCM@FeCNDs

**Figure 10:**
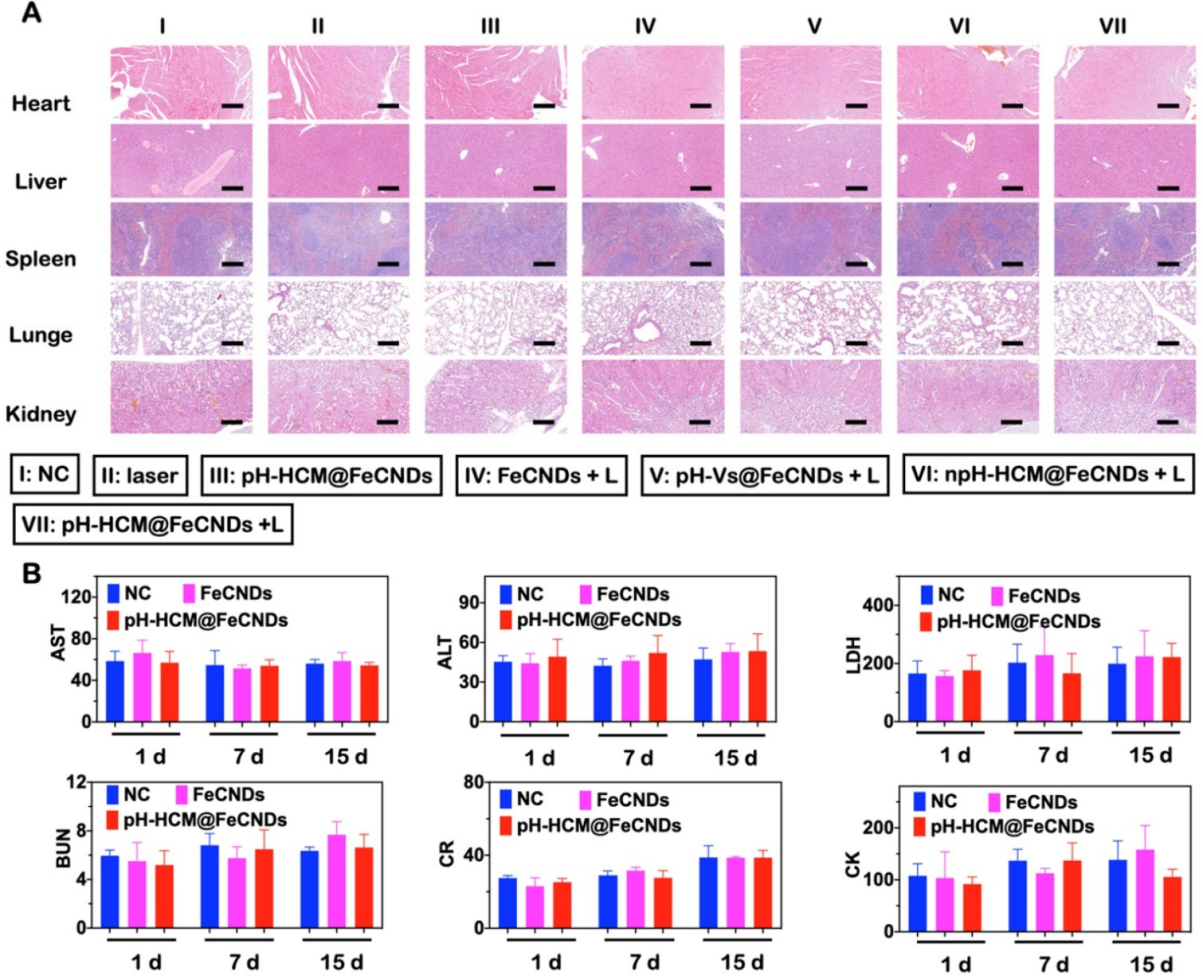
** Toxicity evaluation of pH-HCM@FeCNDs. (A)** H&E staining of main organs collected from mice administrated with different formulations. I: PBS, II: laser, III: pH-HCM@FeCNDs, IV: FeCNDs + laser, V: pH-Vs@FeCNDs + laser, VI: npH-HCM@FeCNDs + laser, and VII: pH-HCM@FeCNDs + laser. Scale bar: 200 µm. **(B)** Blood biochemistry test of BUN, AST, LDH, CR, CK, and ALT at different times after different treatments. The data are expressed as means ± S.D.

The biocompatibility of pH-HCM@FeCNDs was carefully evaluated. As shown in Figure S24, PBS containing red blood cells did not show significant hemolysis after treatment with pH-HCM@FeCNDs in different Fe concentrations (10, 25, 50, 100, 200, and 300 μg Fe/mL) at 37 °C for 3 h. This good blood compatibility was a result of low toxicity of FeCNDs and the camouflage ability of the hybrid membrane, which prevented damage to the red blood cells. Moreover, the change in body weight was negligible during the entire treatment, implying the minimal biotoxicity of these nanomaterials (Figure 8B). Furthermore, no apparent damage was observed in H&E staining images of various organs (heart, liver, spleen, lung, and kidney), indicating minimal short-term toxicity (Figure 10A). The long-term toxicity of pH-HCM@FeCNDs was confirmed via blood biochemical examinations. It was found that compared with healthy mice, the levels of urine nitrogen (BUN), aspartate aminotransferase (AST), lactate dehydrogenase (LD), creatinine (CR), creatine kinase (CK), and alanine aminotransferase (ALT) showed no apparent changes within 15 days post i.v. injection, suggesting low side effects of pH-HCM@FeCNDs (Figure 10B).

## Conclusion

In conclusion, a multifunctional nanoplatform pH-HCM based on pH-Vs and CM was prepared to load FeCNDs nanodots for cancer thermal/chemodynamic treatment. Owing to the appropriate size and functional proteins such as CD62p, pH-HCM@FeCNDs was accumulated in the tumor area by active and passive targeting. Furthermore, the loaded FeCNDs nanodots were released in an acidic environment and were found to penetrate into the deep area because of small size. In the *in vitro* MCS model, pH-HCM@FITC-Dextran possessed higher penetration efficiency (72.84%) under acidic condition than its non-responsive counterparts (17.77%). Moreover, in the *in vivo* fluorescence image experiment, the fluorescence intensity of pH-HCM@IR/Fe was 3.18 times higher than that of pH-Vs@IR/Fe at 24^th^ hour post i.v. injection, indicating a satisfactory target ability of CM. Finally, pH-HCM@FeCNDs showed the best antitumor and antimetastatic effects on orthotropic breast cancer compared with npH-HCM@FeCNDs and pH-Vs@FeCNDs. Thus, owing to the good responsibility, satisfactory targeting ability, and excellent biocompatibility, this study provides novel insights into delivering and releasing ultrasmall nanoparticles based on a hybrid cell membrane.

## Supplementary Material

Supplementary figures and table.
