# Supplementary material for: pH-responsive hybrid platelet membrane-coated nanobomb with deep tumor penetration ability and enhanced cancer thermal/chemodynamic therapy
Source: Theranostics. 2022 May 16;12(9):4250–68. doi: 10.7150/thno.68996 (PMC9169365; doi:10.7150/thno.68996)
Supplement: Supplementary file 1 — Supplementary figures and table. [file thnov12p4250s1.pdf]

Supporting Information

pH-responsive hybrid platelet membrane-coated nanobomb with deep tumor penetration ability and enhanced cancer thermal/chemodynamic therapy

Huang Yang[1], Yuan Ding[1], Zongrui Tong, Xiaohui Qian, Hao Xu, Fenghao Lin, Guoping Sheng*, Liangjie Hong, Weilin Wang*, Zhengwei Mao*

H. Yang, Z. Tong, F. Lin, L. Hong, Prof. Z. Mao

MOE Key Laboratory of Macromolecular Synthesis and Functionalization Department of Polymer Science and Engineering, Zhejiang University, Hangzhou 310027, China

E-mail: zwmao@zju.edu.cn

Dr. Y. Ding, X. Qian, H. Xu, Prof. W. Wang

Department of Hepatobiliary and Pancreatic Surgery, the Second Affiliated Hospital, School of Medicine, Zhejiang University, Hangzhou 310009, China

E-mail: wam@zju.edu.cn

Dr. G. Sheng

Department of Infectious Disease, Shulan (Hangzhou) Hospital Affiliated to Zhejiang Shuren University, Shulan International Medical College, Hangzhou 310022, China

E-mail: gpsheng@zju.edu.cn

H.Y. and Y.D. contributed equally to this work

Name	Essential component	Application
FeCNDs	Fe(III), gallic acid, PVP	nanodots for CDT and PTT
CM	Platelet membrane	endue pH-HCM with active targeting ability
pH-Vs	DSPE-PEOz	negative control of pH-HCM without active targeting ability
npH-HCM	DSPE-PEG, CM	negative control of pH-HCM without responsiveness
pH-HCM	pH-Vs, CM	nanocarrier with responsiveness and active targeting ability
pH-Vs @FeCNDs	pH-Vs, FeCNDs	negative control of pH-HCM@FeCNDs without active targeting ability
npH-HCM @FeCNDs	npH-HCM, FeCNDs	negative control of pH-HCM@FeCNDs without responsiveness
pH-HCM @FeCNDs	pH-HCM, FeCNDs	nanobomb with responsiveness and targeting ability for CDT and PTT
pH-Vs @FITC-Dextran	pH-Vs FITC-Dextran	negative control of pH-HCM @FITC-Dextran without active targeting ability
npH-HCM @FITC-Dextran	pH-HCM, FITC-Dextran	negative control of pH-HCM @FITC-Dextran without responsiveness
pH-HCM @FITC-Dextran	npH-HCM, FITC-Dextran	substitute of pH-HCM@FeCNDs for cellular uptake experiment and penetration experiment
pH-Vs @IR/Fe	pH-Vs, FeCNDs, IR780	negative control of pH-HCM@IR/Fe without active targeting ability
npH-HCM @IR/Fe	npH-HCM, FeCNDs, IR780	negative control of pH-HCM@IR/Fe without responsiveness
pH-HCM @IR/Fe	pH-HCM, FeCNDs, IR780	loading IR780 for in vivo fluorescence image

Table S1. Name, formulation and application of research objects.

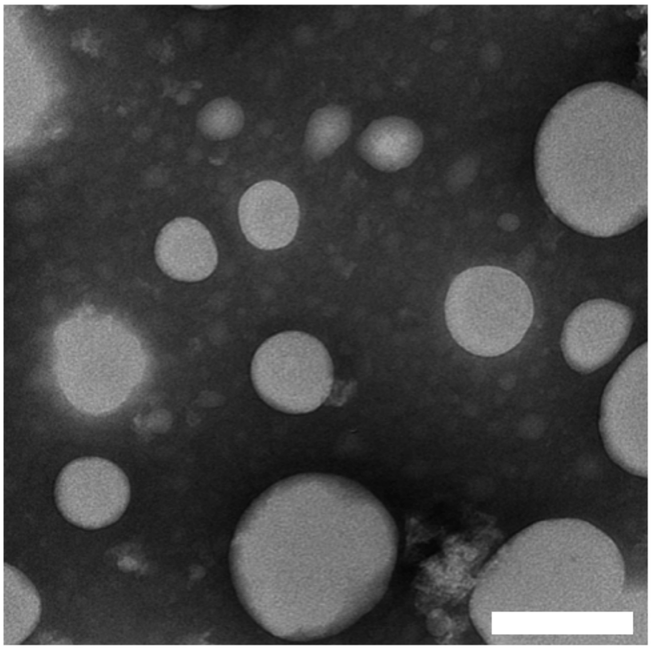

Figure S1. Typical TEM of the vesicles composed of CM after staining with phosphotungstic acid. Scale bar: 200 nm.

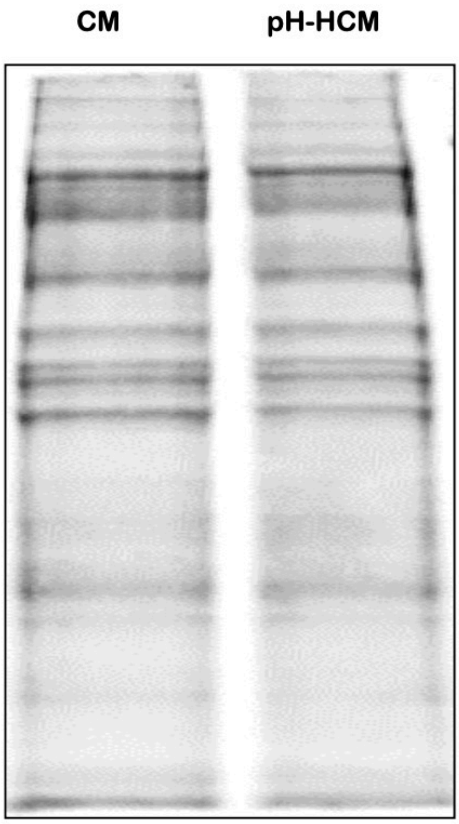

Figure S2. SDS-PAGE analysis of membrane proteins isolated from the CM and pH-HCM.

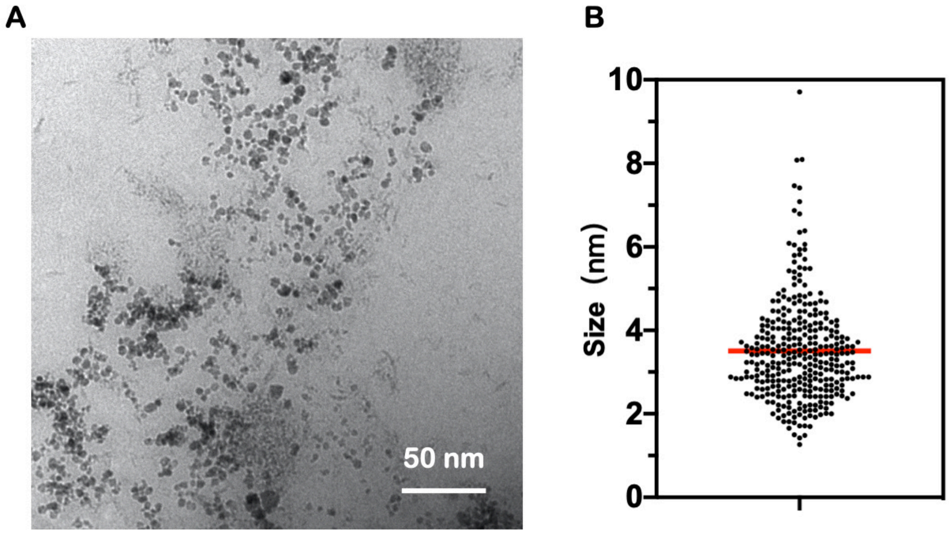

Figure S3. (A) Typical TEM image of FeCNDs nanodots. Scale bar: 50 nm. (B) Mean diameter of FeCNDs nanodots by counting 330 nanodots.

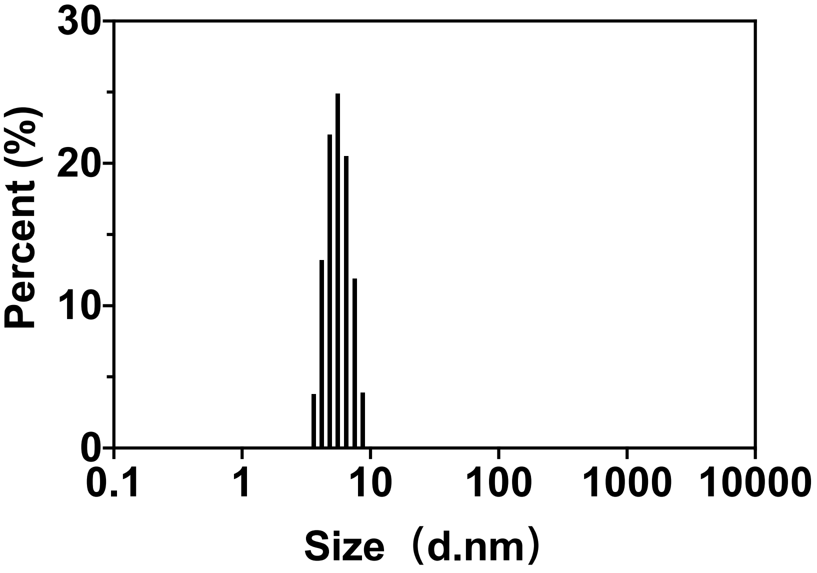

Figure S4. Size distribution of ultrasmall nanoparticles FeCNDs.

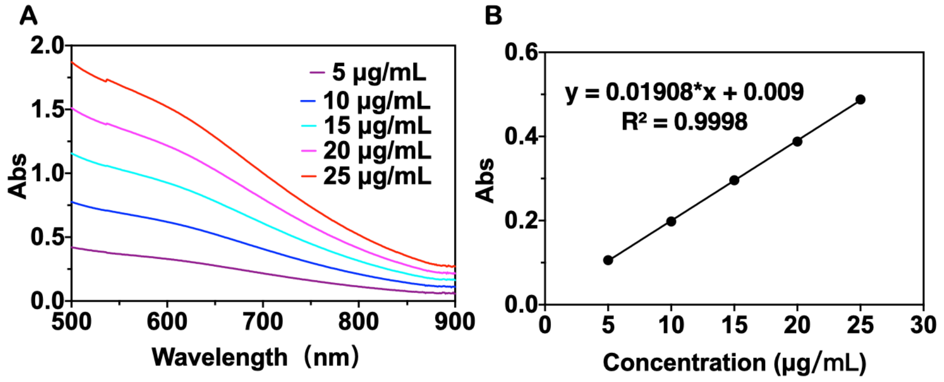

Figure S5. (A) The UV-vis-NIR spectrum and (B) standard curve of FeCNDs solution.

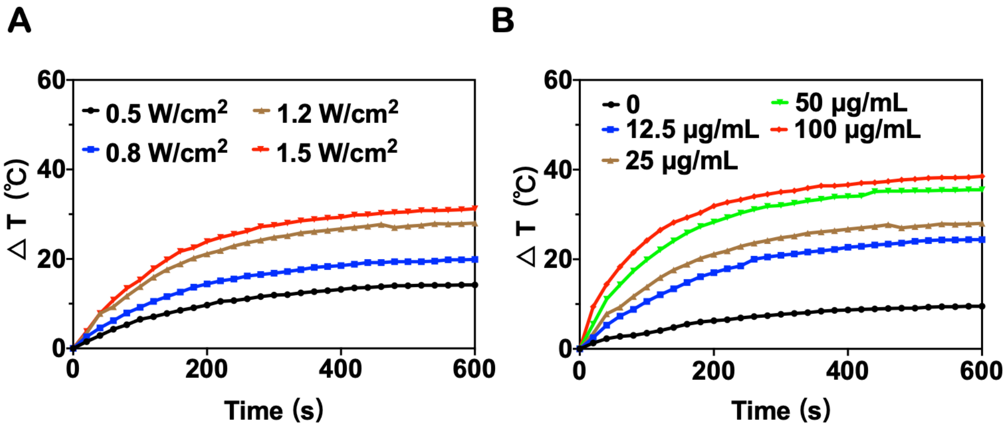

Figure S6. (A) Heating profiles of FeCNDs (25 μg Fe/mL) at different irradiation intensities of 808 nm laser irradiation. (B) Heating profiles of FeCNDs with different Fe concentrations at 1.2 W/cm^2 of 808 nm laser irradiation.

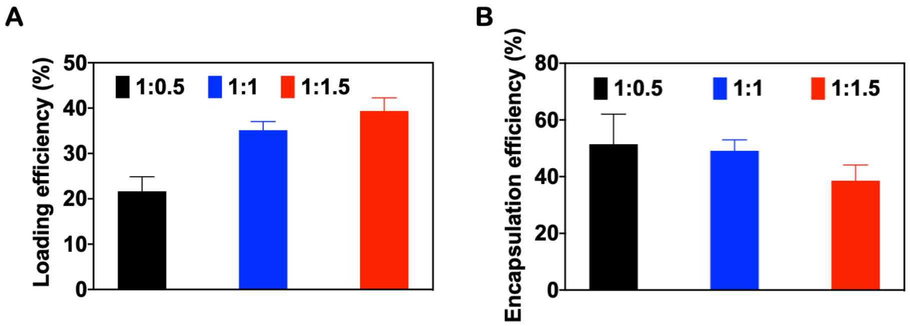

Figure S7. (A) The loading efficiency and (B) encapsulation efficiency of pH-HCM@FeCNDs in the different mass ratios of pH-HCM: FeCNDs

		Density			Mass fraction
FeCNDs	Component	Fe(III)	GA	PVP	39.4%
	Density (g/cm^3)	7.86	1.69	1.44	
	Mass fraction	3.19%	29.15%	67.66%	
	Molar weight (g/mol)	55.845	170.12	8000	
	Molar fraction	24.11%	72.32%	3.57%	
	Density of FeCNDs: 3.16 g/cm^3 (evaluated by molar weighting)				
pH-HCM	1.16 g/cm^3				60.6%
Number of FeCNDs nanodots loaded in each pH-HCM@FeCNDs nanoparticle ≈ 4.89 × 10^3					
Volume fraction of FeCNDs nanodots loaded into the hybrid membrane ≈ 22.39 %					

Table S2. Roughly evaluated number and volumce fraction of FeCNDs nanodots in each pH-HCM on average. The mass percentage of FeCNDs nanodots in pH-HCM@FeCNDs was 39.4% based on ICP-MS result (Figure S7). The density of FeCNDs nanodots was 3.16 g/cm^3, which was evaluated roughly by molar weighting. The density of pH-HCM was 1.16 g/cm^3 based on similar materials. Therefore, the number of FeCNDs nanodots in each pH-HCM@FeCNDs nanoparticle was calculated as 4.89 × 10^3, and the volume percentage of FeCNDs nanodots in hybrid membrane was calculated as 22.39% according to the above data and size of nanodots and nanoparticles.

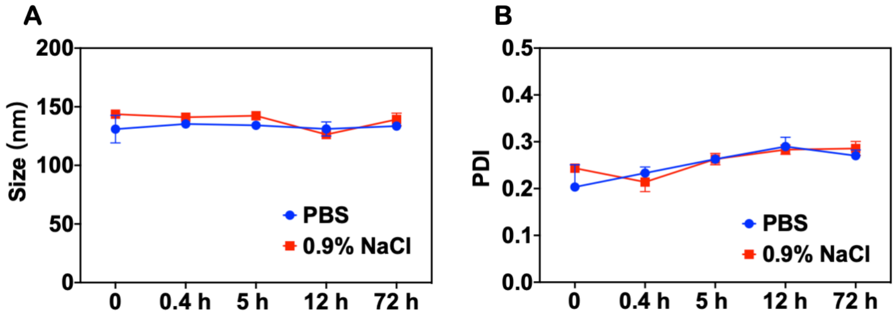

Figure S8. (A) The hydrodynamic size and (B) PDI of pH-HCM@FeCNDs in PBS buffer solution or 0.9% NaCl solution for the different time at 4 °C.

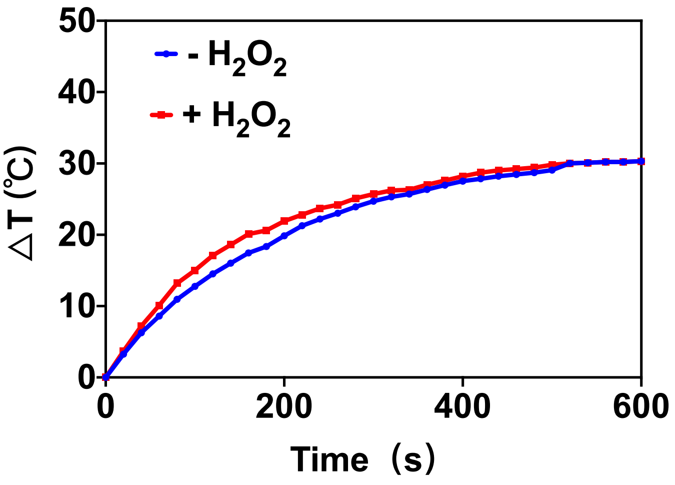

Figure S9. Heating profiles of pH-HCM@FeCNDs (25 μg Fe/mL) with/ without H_2O_2 (10 μM) at 1.2 W/cm^2 of the 808 nm laser irradiation.

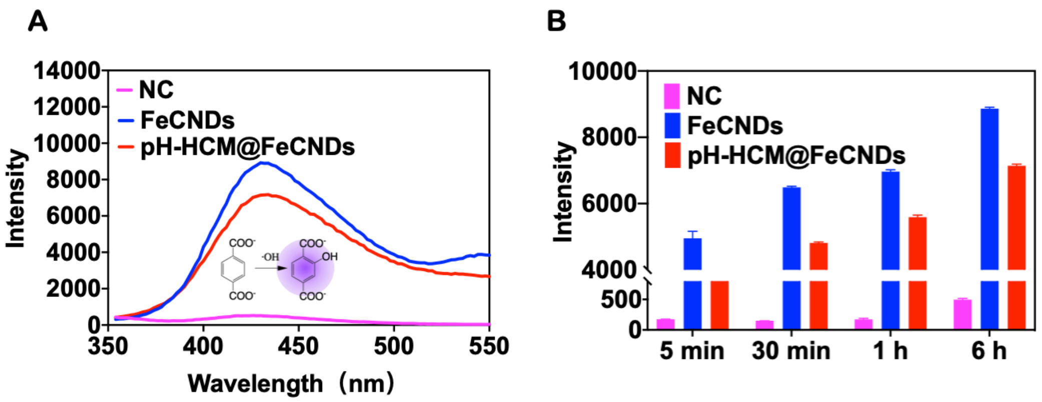

Figure S10. (A) Fluorescence spectra of p-phthalic acid (TA) solution after reaction with H_2O_2, H_2O_2 + FeCNDs, and H_2O_2 + pH-HCM@FeCNDs for 6 h. (B) Fluorescence intensity of p-phthalic acid solution after reaction with H_2O_2, H_2O_2 + FeCNDs, and H_2O_2 + pH-HCM@FeCNDs for 5 min, 30 min, 1 h and 6 h.

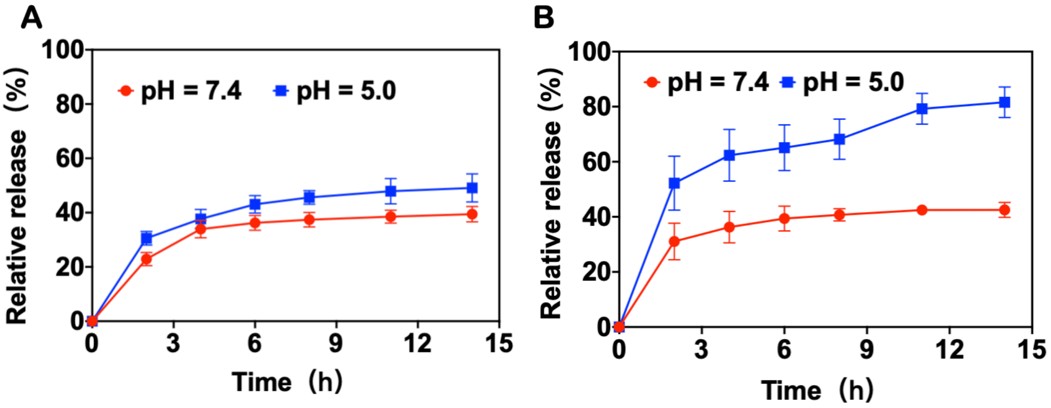

Figure S11. FITC-Dextran released from (A) npH-HCM@FITC-Dextran and (B) pH-HCM@FITC-Dextran at different pH values.

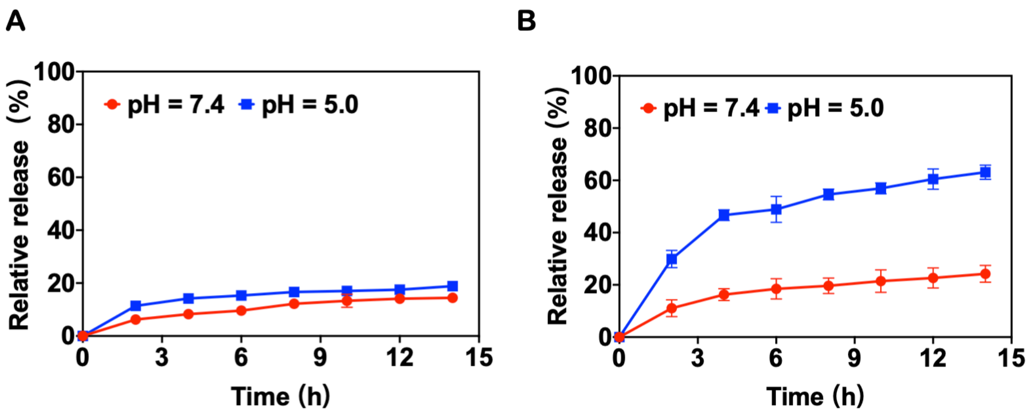

Figure S12. FeCNDs released from (A) npH-HCM@FeCNDs and (B) pH-HCM@FeCNDs at different pH values.

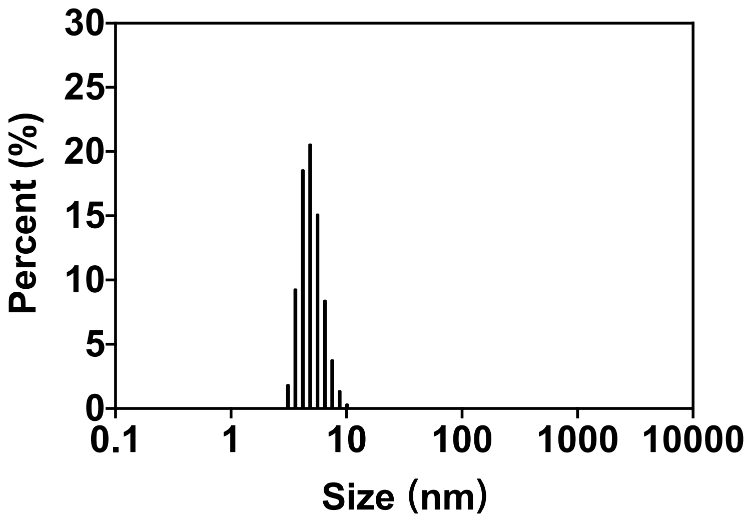

Figure S13. Size distribution of FITC-Dextran

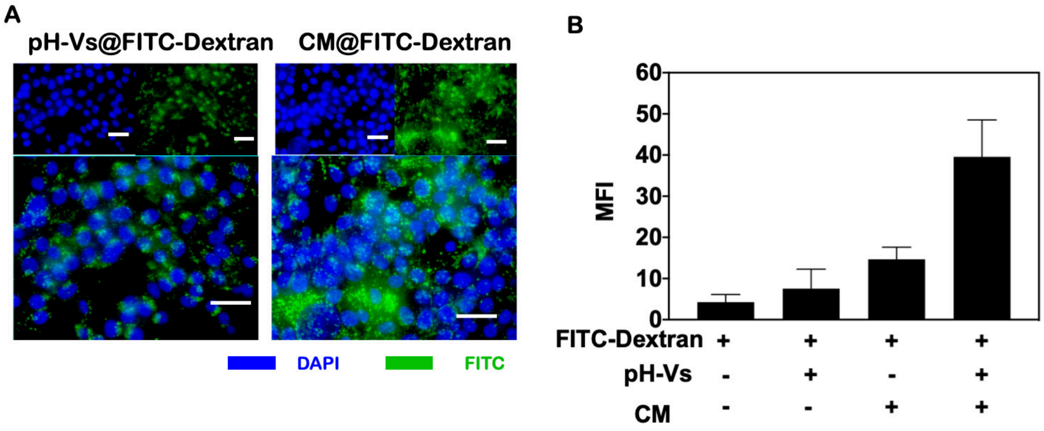

Figure S14. (A) Fluorescence images of 4T1 cells incubated with pH-Vs@FITC-Dextran or CM@FITC-Dextran. Blue fluorescence presents nuclear from DAPI; green fluorescence presents FITC-Dextran. Scale bar: 20 µm. (B) Mean green fluorescence intensity of 4T1 cells after incubation with different formulations.

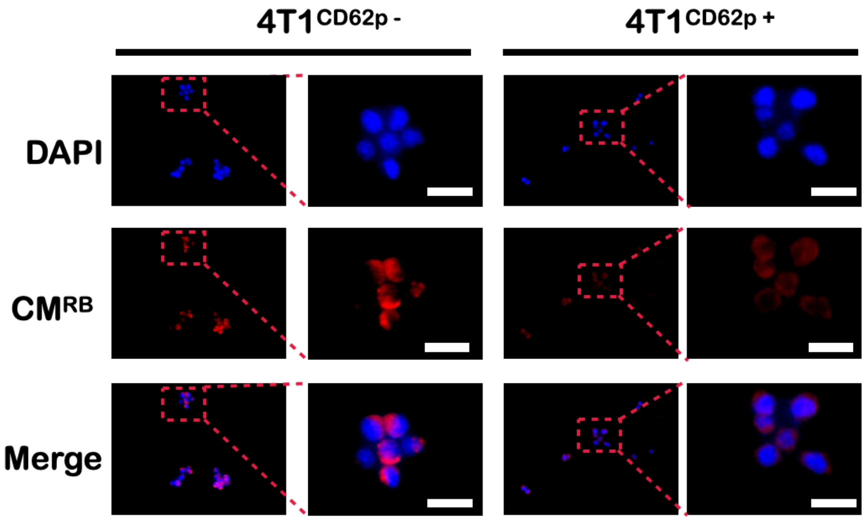

Figure S15. Cellular uptake of rhodamine B-labeled platelet membrane (CM^{RB}) by 4T1 cells pretreated with CD62p ($4T1^{CD62p+}$). 4T1 cells pretreated without CD62p ($4T1^{CD62p-}$) were used as control group. Scale bar: 20 μm.

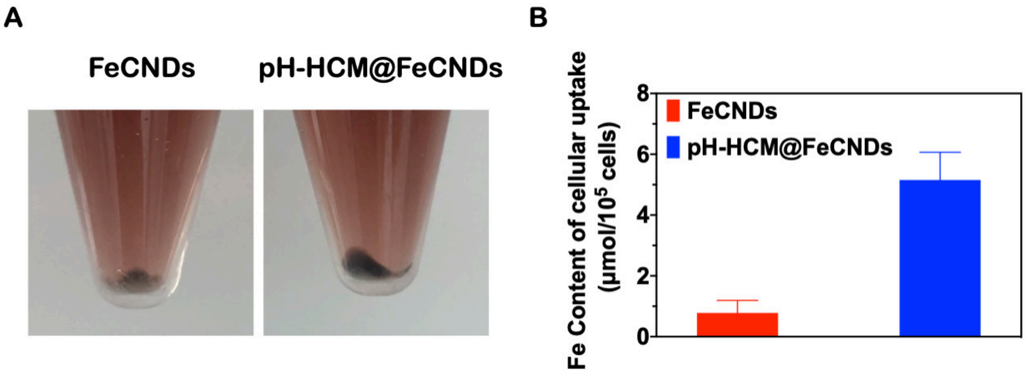

Figure S16. (A) Photographs of centrifugation products of cell suspension after co-incubation of FeCNDs and pH-HCM@FeCNDs. (B) Iron content of cellular uptake when 4T1 cells were incubated with FeCNDs and pH-HCM@FeCNDs (25 μg Fe/mL) for 12 h.

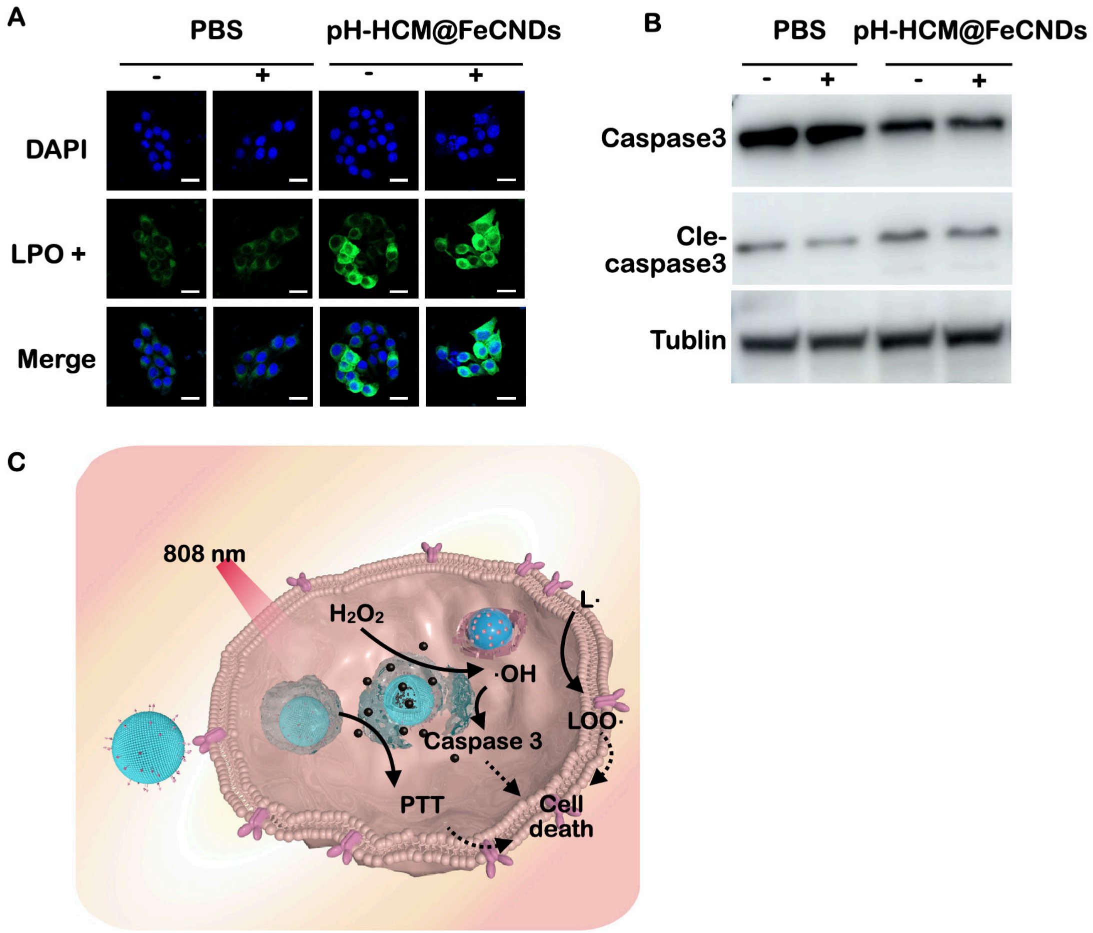

Figure S17. (A) Fluorescence images of 4T1 cells stained with C11-BODIPY 581/591 after different treatments. (B) The protein levels of caspase 3 and cleaved caspase 3 in 4T1 cells after different treatments. The irradiation density was 1.2 W/cm^2, and the irradiation time was 6 min. (C) Antitumor action of pH-HCM@FeCNDs in 4T1 cells. H_2O_2 was catalyzed to hydroxyl radicals (•OH), resulting in cell membrane liposome peroxidation and activation of caspase 3. Meanwhile, high temperature caused by pH-HCM@FeCNDs under 808 nm laser led to thermal ablation of tumor cells. Scale bar: 20 µm.

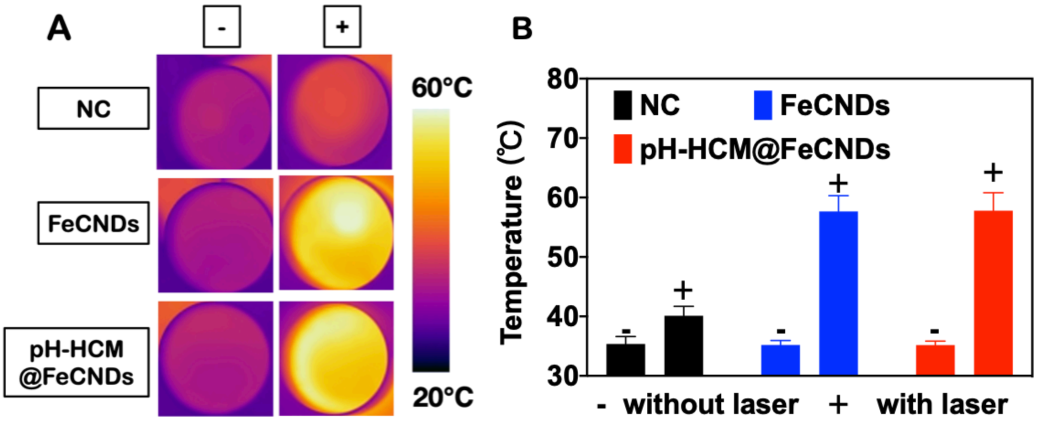

Figure S18. (A) Infrared thermal graphics and (B) temperature of DMEM after different treatment in cell experiment. The Fe concentration of FeCNDs and pH-HCM@FeCNDs was 25 μg/mL. The radiation intensity was 1.2 W/cm^2, and the radiation time was 6 min.

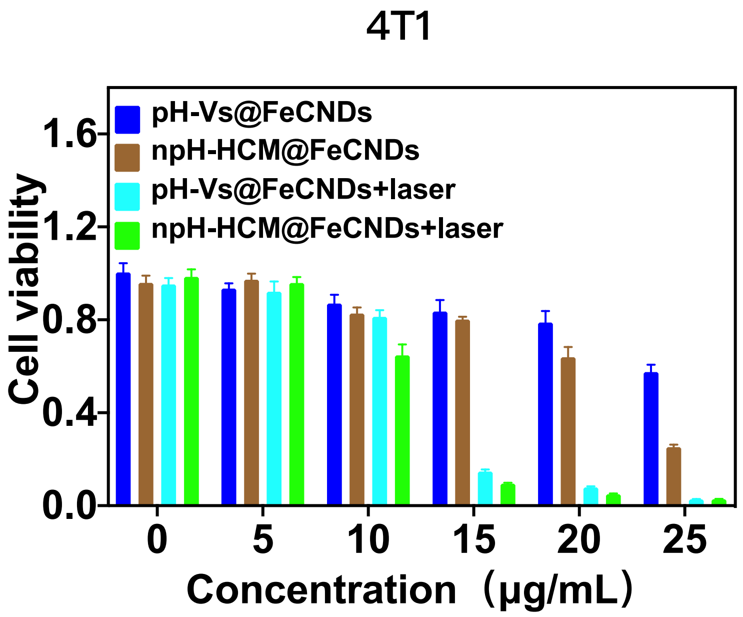

Figure S19. Cell viability of the 4T1 cells incubated with different concentrations of pH-Vs@FeCNDs or npH-HCM@FeCNDs for 12 h with or without the 808 nm laser irradiation (1.2 W/cm^2, 6 min).

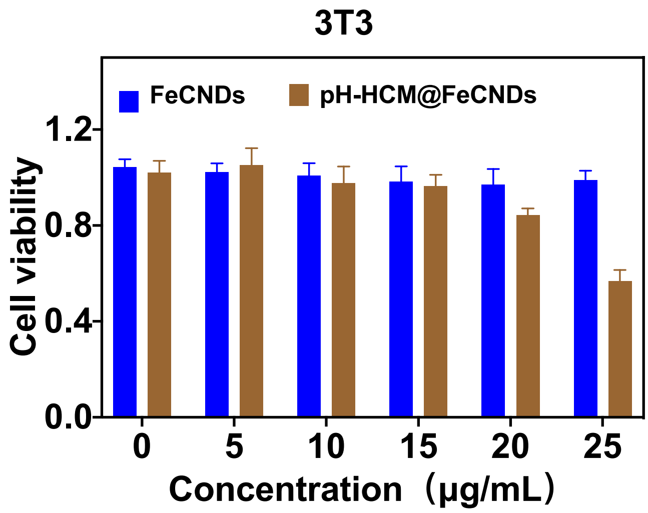

Figure S20. Cell viability of the 3T3 cells incubated with different concentrations of pH-HCM@FeCNDs for 12 h.

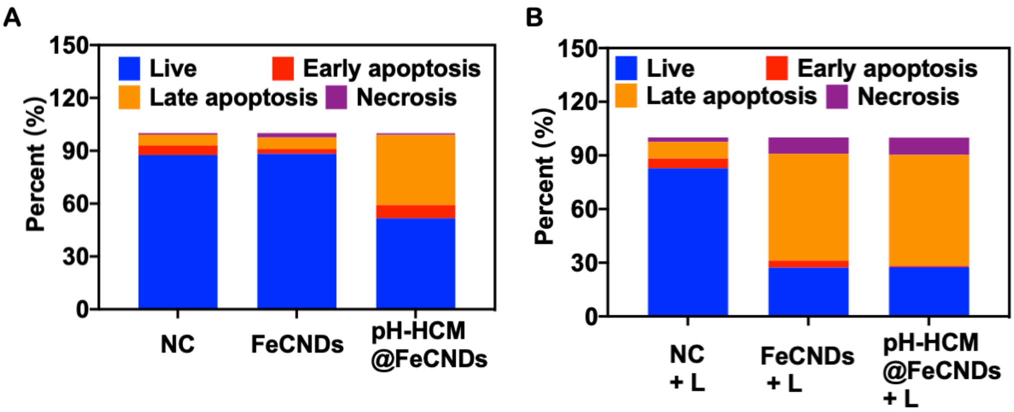

Figure S21. (A) Annexin V/PI analyses of 4T1 cells after treatments with FeCNDs and pH-HCM@FeCNDs. (B) Annexin V/PI analyses of 4T1 cells after treatments with FeCNDs and pH-HCM@FeCNDs with laser. The Fe concentration of FeCNDs and pH-HCM@FeCNDs was 25 μg/mL. The radiation intensity was 1.2 W/cm^2, and the radiation time was 6 min.

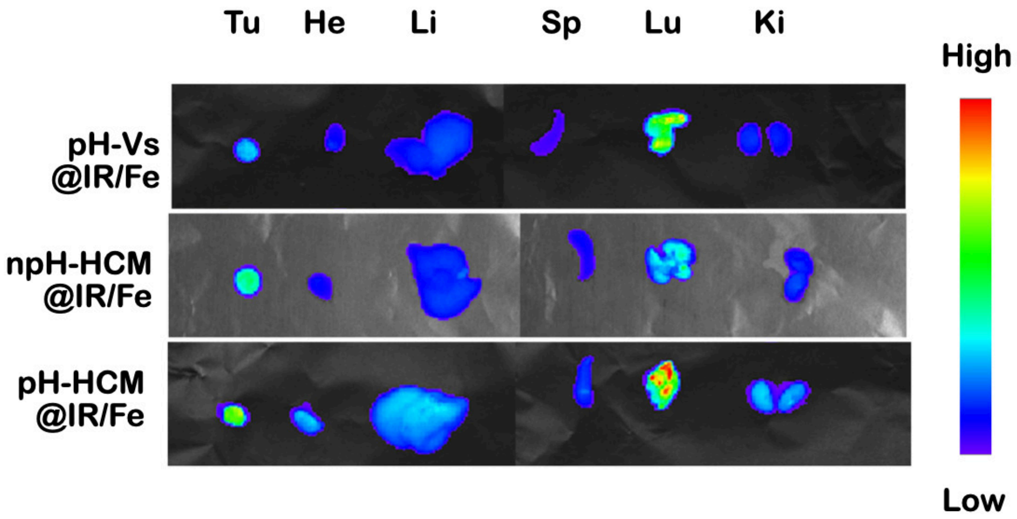

Figure S22. Fluorescence images of tumor and organs (heart, liver, spleen, lung, kidney) at 24th hour after i.v. injection with pH-Vs@IR/Fe, npH-HCM@IR/Fe, or pH-HCM@IR/Fe.

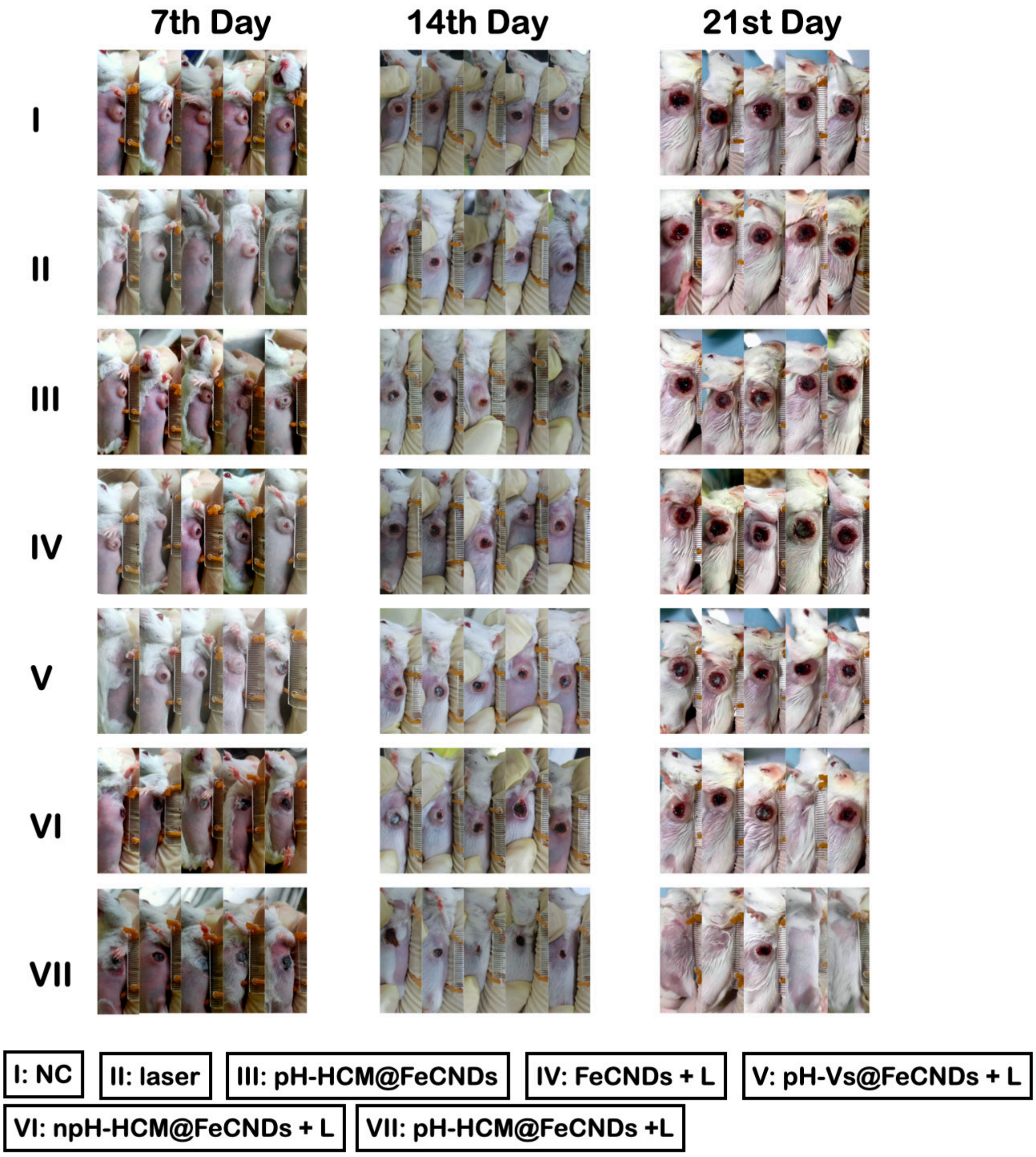

Figure S23. Photographs of tumor-bearing mice at 7th, 14th and 21st day after i.v. injection with different formulations. The irradiation density was 1.2 W/cm^2, and the irradiation time was 6 min. In the first seven days, both group VI and group VII showed similar tumor thermal ablation effect. However, tumor recurrence in four mice of group VI happened from margins of thermal ablation after day 7, while that in only one mouse of group VII happened after day 14.

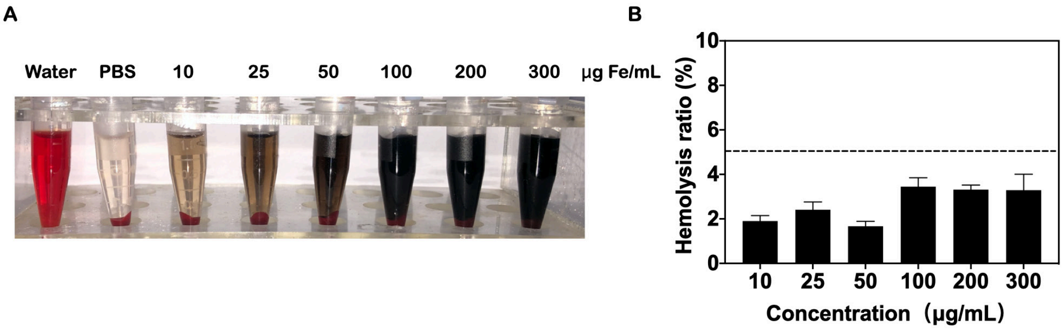

Figure S24. (A) Photograph of hemolysis experiment and (B) relative hemolysis ratios of pH-HCM@FeCNDs in different concentrations.
